# A preliminary study of facial soft tissue thickness for forensic facial reconstruction in a Nigerian adult female community

**DOI:** 10.1007/s00414-026-03794-1

**Published:** 2026-05-25

**Authors:** Nurudeen Adegbite, Manuela Mura, Haliru Shafiu, Christopher Avery, Mohammed Al-Khafajiy, Waqar Ahmed

**Affiliations:** 1 School of Engineering and Physical Sciences, University of Lincoln, Brayford Pool Campus, Lincoln, LN6 7TS UK; 2 Usmanu Danfodiyo University and Teaching Hospital, Garba Nadama Road, Sokoto, Nigeria; 3 Department of Oral and Maxillofacial Surgery, Leicester Royal Infirmary, Leicester, LE1 5WW UK

**Keywords:** Forensic facial reconstruction, Hausa adult female, Facial soft tissue thickness data, Computed tomography, Facial reconstruction

## Abstract

Forensic facial reconstruction is the reproduction of a deceased person’s antemortem face, most commonly achieved using population-based data of facial soft tissue thickness. Facial soft tissue thickness (FSTT) data have been documented for many populations. However, there are no published data for Nigerian female adults. This study measured the facial soft tissue thickness of 32 subjects based on computed tomography scans. Measurements were taken from 50 facial soft tissue thickness points, including 12 mid-sagittal and 19 bilateral (right and left) points, across a diverse age range of 18 to 95 years. The Nigerian female adult consistently shows marginal increase in soft tissue thickness on the left side of the face when compared with the right, with the greatest relative difference at the zygomatic arch and mid-lateral orbit. When compared with the published data of populations in South Africa, Turkey, Korea, and Belgium, there were similarities at the midline but appreciable differences at the lip and chin regions. The right side of the face of the Nigerian female adults was also compared with other populations; the differences at the lateral side of the face were greater than the midline differences. These differences reflects the greater thickness of bilateral soft tissue. This study demonstrates that although the data between different populations are similar, there are still measurable differences at several points. This new dataset will be most applicable for forensic facial reconstruction in Nigerian female adults.

## Introduction

 Forensic facial reconstruction (FFR) is the reproduction of the face of the deceased person from their skull. This is commonly achieved by building the soft tissues onto the skull. This practice has been an aid in recognition since the 1370 BC (Before Christ) Egyptian burial ceremony as a death mask [1]. This technique also existed during the 2nd and 3rd centuries BC (Before Christ) as part of Roman entombment rituals and in the bronze portrait age in the passé Christian era [1].

Different methods for forensic facial reconstruction have been used. This can broadly be classified into two techniques: two-dimensional [1, 2] and three-dimensional [1–3]. These techniques are either manual or computer-based. The standard practice of FFR is the reproduction of the face conventionally by using the facial soft tissue thickness (FSTT) dataset derived from the same population.

Typically, facial soft tissue thickness (FSTT) is measured at defined anatomical points from the skull [1–9] and usually with the skull placed in the Frankfurt position [1, 3–10]. The Frankfurt position is the horizontal plane passing between the lowest point on the margin of the orbit (Ro) and the highest point on the brim of the auditory meatus (Po). The measurements are usually taken at 90° from the defined soft tissue points on the skull to the skin [1, 11, 12].

However, there is no consensus on an optimal number of FSTT points for forensic facial reconstruction. A range of points have been used in published works, varying from as low as 9 [1] to 60 [13]. Most authors have at least studied midline facial soft tissue thickness [14, 15], but commonly a combination of midline and lateral points [4–6, 13]; this combination will produce a wider coverage of the face, although not all published works stated which side of the face was studied.

Facial soft tissue thickness are a variable of several factors including population affinity [1, 2, 4–6, 14–26], sex [1, 2, 13], age [1, 27] and body mass index (BMI) [1, 2, 5, 14–20, 27], Table 1. Reports have shown that dental malocclusion is also a significant factor [35]. There are appreciable differences in facial soft tissue thickness values between different populations; hence, population-based values are often used (Table 1) [1, 2, 13, 22, 36]. Some authors have argued that the slight millimetre differences are of no practical significance [8], and others believe the differences in the rendered appearance are due to the underlying population-based bony differences.

**Table 1:** Defining facial soft tissue thickness as seen in previous studies

Study name (abbreviation)	Adult population studied	M/F	Image (scan)/number of FSTT points	Number of subjects, age brackets (years)	BMI as stated
Hwang et al. [28]	Koreans	M/F	Cone-beam computed tomography/31	100 (M = 50, F = 50)/20 to 36	
Ruiz [18]	Colombians	M/F	Cone-beam computed tomography/17	30 (M = 26, F = 4)/18 to 35	
Lodha et al. [29]	Gujarati, Indians	M/F	Computed tomography/25	489 (M = 324, F = 165)/17 to 65	
Moritsugui et al. [30]	Brazilians (mid-west region)	M/F	Cone-beam computed tomography/21	101 (M = 45, F = 56)/18 to > 41	
Sahni et al. [5]	Indian (north-west)	M/F	Magnetic resonance imaging/29	300 (M = 173, F = 127)/18 to 70	
Eftekhari-Moghadam et al. [14]	Iranian (south-west)	M/F	Magnetic resonance imaging/	100 (M = 160, F = 63)/18 to 50	
Bulut et al. [2]	Turkish	M/F	Computed tomography/31	320 (M = 160, F = 160)/18 to 80	< 20, > 20 – <25, > 25 (L, N, OB)
Manhein et al. [31]	Black and White Americans/midline and right	M/F	Ultrasound/19	197, 19 to 55	
Deng et al. [13]	Yangtze River delta Han (China)	M/F	Cone-beam computed tomography/60	424 (M = 130, F = 294)/21 to 50	
Guyomarc’h et al. [20]	French (France)	M/F	Computed tomography/37 (Reported no difference btw m/f)	500 (M = 265, F = 235)/18 to 96	< 25, > 25 (N, OV)
Navic et al. [32]	Thai (Thailand)	M/F	Needle puncture technique (12 h since death)/27	100 (M = 50, F = 50)/51 to 95	
Tedeschi-Oliveira et al. [23]	Guarulhos, São Paulo (but of mixed ethnicity) (Brazil)	M/F	Needle puncture technique (12 h since death)/32	40 (M = 26, F = 14)/17 to 90	< 20, > 20 – <25, > 25 - <29.9, > 30 (obese)
Codinha et al. [19]	Lisbon, Portuguese	M/F	Needle puncture technique (24 h since death)/20 (8 midline & 12 bilateral)	151 (M = 103, F = 48)/20 to 99	< 20, > 20 – <25, > 25 (L, N, OV)
De Greef et al. [33]	Belgium	M/F	Ultrasound/52	967 (M = 457, F = 510)/20 to 99	< 20, > 20 – <25, > 25 (L, N, OB)
Coskun et al. [34]	Greek	M/F	Computed tomography/22 (5 midline, 17 laterally)	100 (M = 50, F = 50)/18 to 99	
Domaracki and Stephan, [24]	Australian	M/F	Sooted needle puncture technique (embalmed for > 6 months)/13 (7 midline, 3 bilaterally)	33 (M = 19, F = 14)/46–92	

Abbreviations defined; M=male, F=female, L=lean, N= normal, OB=obese, OV= overweight, BMI= body mass index

Different body mass index classifications have been used in FSTT studies (Table 1). Historically, researchers have measured weight rather than body mass index, though in the last decades, most FSTT studies have focused on body mass index [31, 33] rather than weight. Some authors have used only subjects with a normal body mass index (BMI) [37, 38], while others have compared the normal BMI to the overweight subgroup [2, 33]. However, some studies did not investigate body mass index [39], probably because this criterion will increase the number of subjects required. Still, most published literature agrees that facial soft tissue thickness values will increase with BMI increase [1, 2, 36]. However, conflating the facial thickness of the deceased with BMI values may be challenging because BMI may not always be constant.

There is a significant variation in the age ranges in FSTT studies. Publications from Africa have used age ranges in years, such as 20 to 35 [26, 37], 18 to 35 [35, 39], 12 to 71 [40] and 17 to 33 [41] (Table 1). Studies from other continents have used either similar or broader age ranges, such as 20 to 36 [28], 17 to 65 [29], 18 and > 41 [30], 18 to 70 [5] and 18 to 80 [2] (Table 1). There are a lot of variation in the literatures on the effect of age on facial soft tissue thickness. 

Sex has also been shown to be a co-determinant of facial soft tissue thickness value [1, 2, 9]. Authors have studied both sexes differently [2, 5, 20, 26, 33, 37, 41] for comparison; some other researchers combined both sexes to generate a single dataset (Table 1). Others have studied either males [26] or only females [39].

Different imaging and measurement techniques have been used in FSTT studies. Historically, facial soft tissue thickness was determined manually from cadavers, but results were inaccurate due to inherent difficulties in palpating for reference points and further complicated by variable shrinkage of tissues [24, 32, 38]. More recently, cadaveric studies have used freshly deceased individuals of less than 12 [38] or 24 h [32]. Further to cadaveric studies, different imaging modalities have been used in measurements of facial soft tissue thickness (Table 1).

Initially, measurements were from cephalometric X-ray by using acetate tracing and a pencil [35]. However, most contemporary studies have used modern imaging of living subjects with magnetic resonance imaging (MRI) [9, 14, 15, 17], ultrasound [33, 37, 42], computed tomography (CT) [1, 2, 4, 29, 34, 39, 40, 43] and most recently cone beam computed tomography (CBCT) [13, 18, 28, 30], Table 1. CT and MRI both give good results. The measurements of FSTT from live scans are manipulated with specialised software packages such as the image tool in Centricity [39], Skull Measure (CyberMed) [28], Amira [2, 34] and iCAT Vision [18].

The number of subjects analysed in facial soft tissue thickness studies has varied considerably, with the highest number reported by De Greef et al. (967) [33], Manhein et al. (907 of 551 children and 256 adults) [31] and Guyomarc’h et al. (500) [20]. Recently, Shehata et al. in 2023 studied 10 males and 10 females [41], and Ruiz reported on 26 males and 4 females [18] (Table 1). The reduced number of subjects may reduce accuracy; however, the accurate imaging and proficient measurements of FSTT with software packages should give more precise results than cadaver studies.

In age classifications, the number of subjects within each age bracket will vary considerably and may be very low; Manhein et al. had 3 subjects in the 35 to 45 age brackets for black adult males of normal weight [31], whilst Coskun et al. had 8 male and female subjects in the 18–34 age range and 5 in the 35–44 age brackets [34], Table 1.

At the time of this study, the authors were unaware of any published facial soft tissue thickness data on the Nigerian female adult population. This study aimed to establish a dataset and analyse the impacts of age, sex, and body mass index (BMI) to allow comparison with data for the Nigerian adult male [43] and other populations. This sex specific FSTT data will enhance forensic facial reconstruction and improve identification of human remains. This is a significant concern because of the ever-increasing death toll of people trying to migrate from Africa to Europe.

## Method and materials

### Ethical approval

This study was based on a computed tomography scan with ethical approval from the University of Lincoln, United Kingdom. The authors fulfilled the standards for medical research as stated in the October 2013 principles of Helsinki by the World Medical Association and of the Nigerian Data Protection Regulations (NDPR) for public institutions.

### Computed tomography imaging

In this study, computed tomography scans were performed on live subjects over 7 months, from August 2023 to February 2024, at the Uthman Dan Fodio University in Sokoto, north-western Nigeria. The computed tomography machine is Revolution Apex by General Electric (GE), a 16-slice helical scanner manufactured by Hangwei Medical Systems Co., Ltd.

All subjects were scanned in a supine position with the head centred by equal support to both the left and right sides of the face. Those that were not well-centred were rejected as the measurement could not be optimised. Each subject had axial, sagittal and coronal views analysed. The images were acquired at 2.5 mm thicknesses, and the data were manipulated to create a 1.25 mm high spatial resolution slice.

There was no additional radiation risk to any subject because the images were required for purposes unrelated to this study. Images were selected based on the exclusion criteria stated under data collection below. A shared online link was created at the University of Lincoln, and suitable images were remotely downloaded for analysis by a maxillofacial radiologist of the Uthman Dan Fodio University, Sokoto, Nigeria.

### Ethnicities studied and data collection

The ethnic groups studied included the Hausa, Fulani, Yoruba, and Dakarkari, with the Hausa and Fulani being the predominant ethnic types in this part of the country. The biodata gathered from each subject included date of birth, sex at birth, ethnicity, body mass index, and date of imaging. A standardised Form F was used for each subject dataset collection. All data were anonymised, but with unique identifiable numbers. Exclusion criteria were the same as in previous studies of facial soft tissue thickness [2, 39, 43] and were equally stated on the Form F. Excluded subjects included those with a history of facial swelling, head and neck trauma, orthodontic treatment, bony abnormalities, orthognathic surgery, facial identification marks and cleft lip and palate. And also, those with peculiar tribal marks on the face, such will distort the soft tissues [43, 44]. 

### Body mass index classification

The body mass index was determined using the World Health Organisation (WHO) formula, weight (in kg)/height^2 and classification for 18 years and above, as stated thus: less than 18.5 (underweight), 18.5–24.9 (normal weight), 25–29.9 (pre-obese), and equal to 30 or greater (obese). Since the onset of facial soft tissue thickness studies, BMI/weight has always been reported as an index [45]. In this Nigerian population, normal weight is quite prevalent rather than underweight, overweight, or obesity [46]. 

### Facial soft tissue thickness points defined

In this study, 12 midlines and 19 bilateral facial soft tissue points were defined, making a total of 50, as a standard for each subject. These points are a reproduction of points stated in the study on the combined Nigerian male population (CNME) [43] and listed in Tables 2 and 3.

**Table 2:** Midline facial soft tissue thickness points

FSTT points/(Acronyms)	Definitions	Synonyms
Vertex (v)	The highest point on the head	
Supraglabella (sg)	The most anterior midline points on the forehead	Ophryon (of)
Glabella (g)	The most prominent point between the supra-orbital ridges in the midsagittal plane	
Nasion (n)	Midpoint of the suture between the frontal and the two nasal bones	
Mid-Nasal (ns)	The midpoint of the nasal bone, which is between the nasion and rhinion	
Rhinion (rh)	The end of the nasal bones at the cartilage-bone junction	End of the nasal
Midphiltrum (mp)	Midpoint of the philtral column- located by the length from the midline and midway point between prosthion and the subspinale. Measured from the deepest point (point A) of the maxillary alveolar bone	
Upper lip border (ls)	The most anterior midpoint of the upper vermilion line is measured from the prosthion. Measured from between the central incisors at the level of the cementum–enamel junction to the vermillion border The prosthion is the midpoint between the upper central incisors at the level of the cementum–enamel junction.	Supradentale or alveolare or Labiale superius
Lower lip border (li)	The midpoint of the lower vermilion line. Measured from between the lower central incisors at the level of the cementum–enamel junction to the most anterior of the vermillion of the lower lip	Infradentale or Labiale inferius
Labiomental (lm)	The midpoint of the labiomental groove. The deepest midline point furrow on the mandible between the teeth and the chin prominence	Chin-lip fold sublabiale or supramentale
Mental eminence (me)	The most anterior midpoint of the chin	Pogonion
Menton (mn)	The lowest medial landmark beneath the chin	Gnathion or Beneath the chin

**Table 3:** Bilateral facial soft tissue thickness points

FSTT points/(Acronyms)	Definitions	Synonyms
Frontal eminence (fe)	Centred on pupils’ most anterior projections at both sides of the forehead. A point on the projections at both sides of the forehead (centred on the pupils/lies above the midpoint of the eyebrow). It is located as the most protruding point of the frontal bone along the axis of a line (drawn from ecthocanthion to endocanthion) that vertically bisects the orbit.	
Supraorbital (os)	Above the orbit, it’s the highest point of the upper margin of the orbit (centred on the pupil). Located from a point midway between the ecthocanthion and endocanthion, and by drawing a perpendicular line up to the supraorbital margin	
Infraorbital (io)	The lowest point on the lower margin of the orbit. Located from a point midway between the ecthocanthion and endocanthion, and by drawing a perpendicular line up to the Infraorbital margin.	Orbitale (or) suborbital
Supraglenoid (sg)	This is the root of the zygomatic arch. It is anterior and superior to the external auditory meatus.	
Zygomatic arch (zy)	The most lateral point of the zygomatic arch.	Zygion
Lateral Orbit (lo)	This is an anterolateral point on the zygomatic bone. It is the lowest point on the suture between the maxillary and zygomatic bones. It is lined up vertically to the lateral orbital margin on the zygomatic bone.	
Lateral Glabella (lg)	A point on the medial orbit at the junction of the frontal, maxillary, and lacrimal bones	
Lateral nasal(ln)	A point on the side of the nose in line with the endocanthion. It is a point on the Frankfurt horizontal plane at the side of the bridge of the nose.	
Inferior malar (Im)	The most protruding point of the maxilla lies beneath the zygomatic process at a point on a vertical line with the infraorbital and supraorbital points. It is centred on the vertical line from the pupil and just inferior to the zygomatic process.	Lateral maxilla
Lateral nostril (lno)	This is a point just next to the most lateral point of the alar border.	
Supra-canine (sc)	This is a point on the superior alveolar ridge of the upper canine. It is on the horizontal level of the mid-philtrum and lines up vertically with the cheilion. It is measured from the maximum bulge of the upper canine prominence.	
Infra-canine (ic)	This is a point on the inferior alveolar ridge of the lower canine. It is vertically lined up with the cheilion, on the horizontal level of the chin–lip fold.	Subcanine
Mid-lateral orbit (mlo)	This is a point just next to the lateral orbital border	
Supra-M2 (sm2)	This is a point on the superior alveolar ridge of the second upper molar.	
Infra-M2 (im2)	This is a point on the inferior alveolar ridge of the second lower molar	
Mid-masseter (mm)	A point located halfway between the supraglenoid and the gonion, located about the middle of the masseter, (*located from a coronal view*)	
Gonion (go)	The most lateral point at the angle of the mandible (located on the angle of the mandible),	
Occlusal line (ol)	A point on the anterior part of the ascending ramus in alignment with the line where the teeth occlude anterior laterally	
Mid-mandible (mdm)	A point on the inferior border of the mandible, vertically lined with the supra-M2 (Bulut et al., 2018)	

**Fig. 1:**
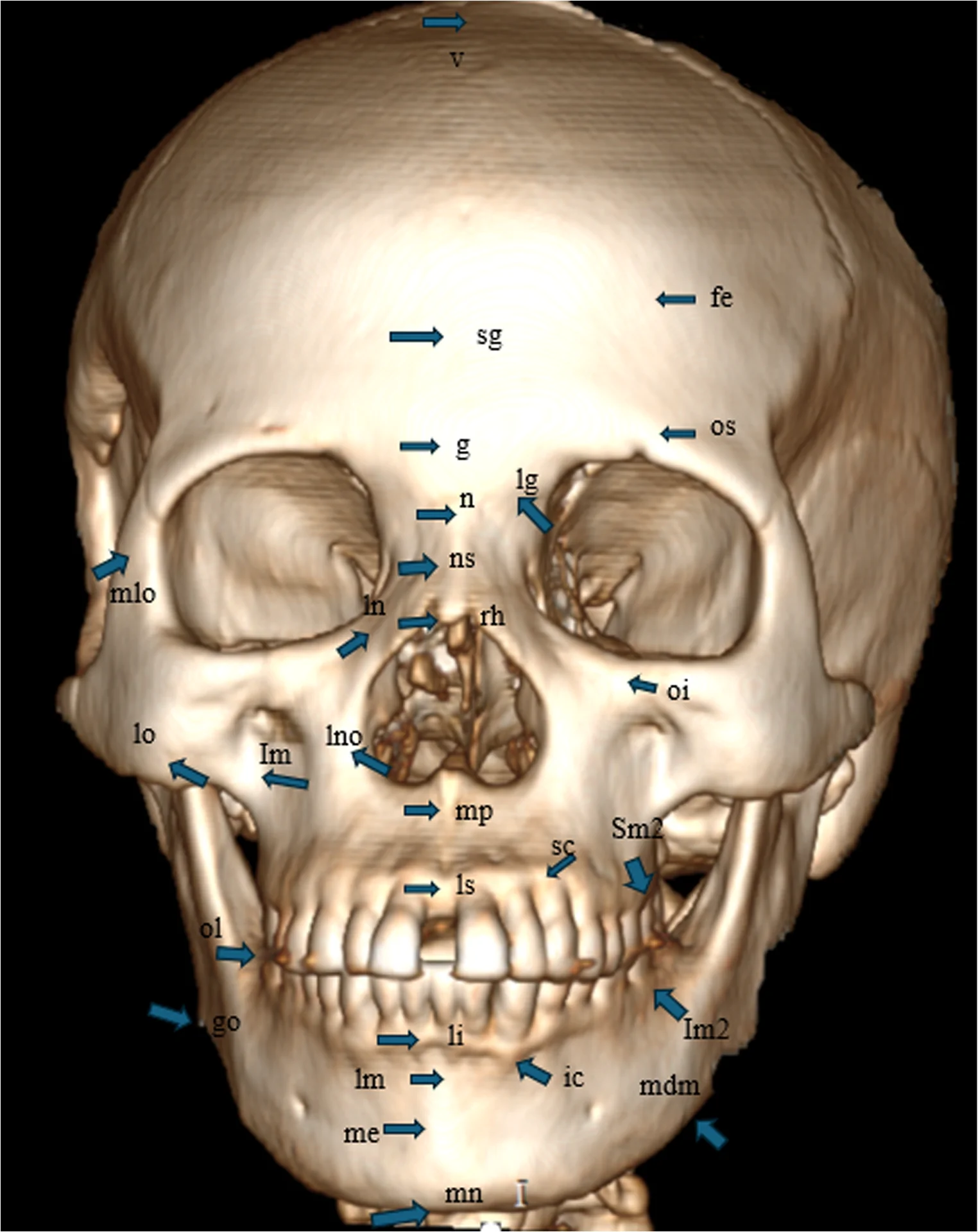
Midline and bilateral facial soft tissue points, defined by the tip of the blue arrowhead

**Fig. 2:**
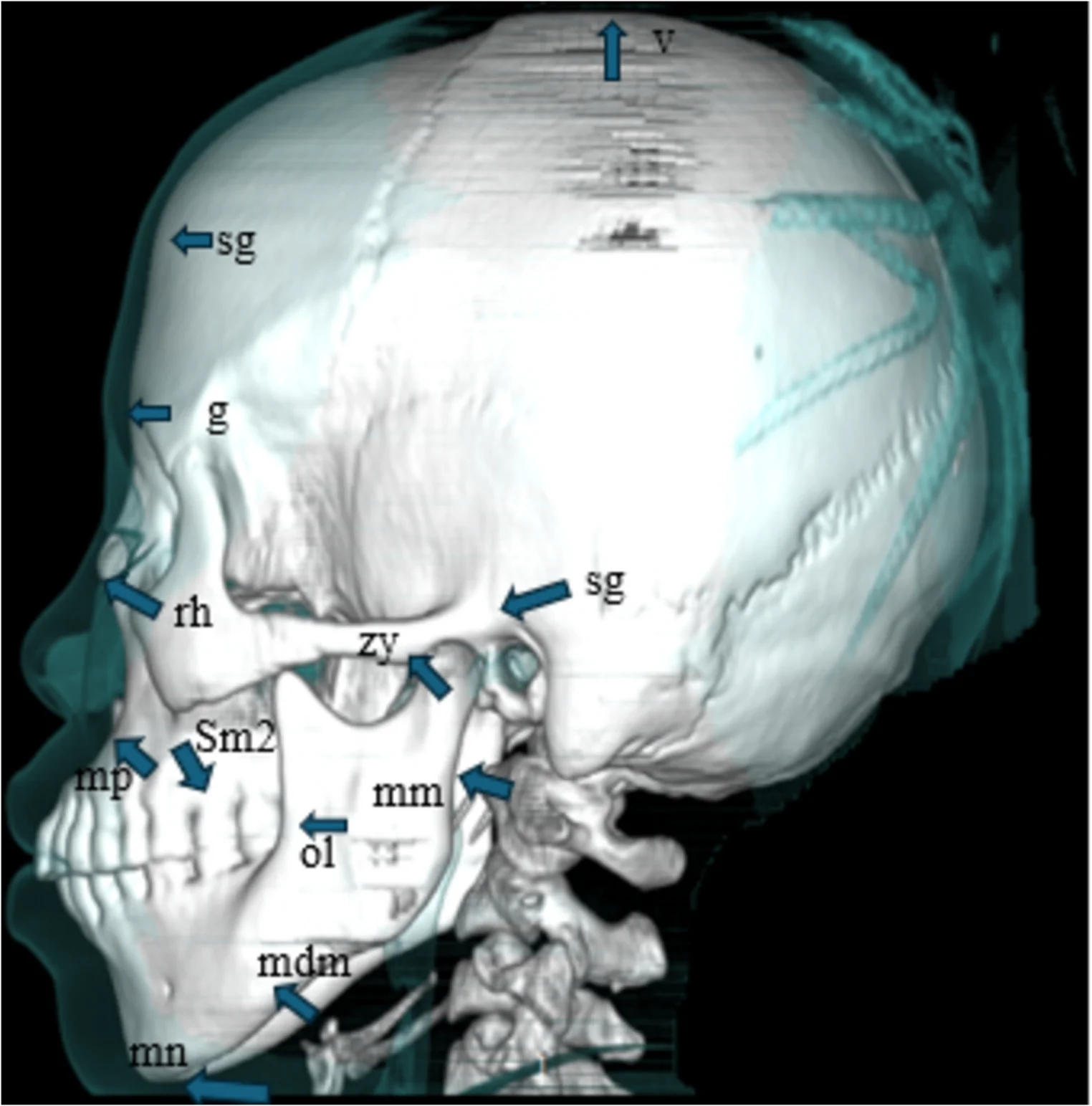
Facial soft tissue thickness points in a lateral bony window, defined by the tip of the blue arrowhead

These points are also replicas from previous studies, including Bulut et al. [2], Wilkinson [1], Sahni et al. [5], Auslebrook et al. [26], De Greef et al. [33], and Cavanagh and Steyn [39]. These points are illustrated in Figs. 1 and 2; the tip of the arrowhead defines each FSTT point.

### Computed tomography scans processing and FSTT estimations

The software package, RadiAnt, was used to estimate all facial soft tissue thickness (FSTT) values. This software is a Medixant Company, PACS DICOM (Digital Imaging and Communications in Medicine) viewer, version 2023.1 (64-bit) [47]. This software demonstrates both the soft and hard tissue windows and is described in detail in the combined Nigerian adult male study [43].

The RadiAnt software supports all types of DICOM images, including monochromatic (e.g., chest x-ray (CXR), computed tomography, colour (for example 3D reconstructions), static images (e.g. CXR), magnetic resonance imaging, ultrasound and Positron Emission Tomography (PET). It rapidly reconstructs images in multiple orthogonal planes (sagittal, axial, coronal, oblique). There is a 3D VR (volume rendering) tool, which allows for soft or hard tissue anomalies to be detected. This software angle tool allows for facial soft tissue thickness to be determined at a 90° angle from bone to skin surface. 

The protocols involved downloading each scan from the shared online drive into RadiAnt. The desired facial soft tissue thickness point was then located on the relevant orthoslice. The measurement at each point was obtained at 90° from the bone to the skin surface with the skull in a Frankfurt position, this is similar to previous studies [1, 2, 39, 40, 48]. The midline facial soft tissue thickness points were located and estimated on the mid-sagittal view using the nasal septum and dentition as guides. Bilateral FSTT points were located on the axial view orthoslice except for the mid-masseter point, which was estimated on the coronal view. 

**Fig. 3:**
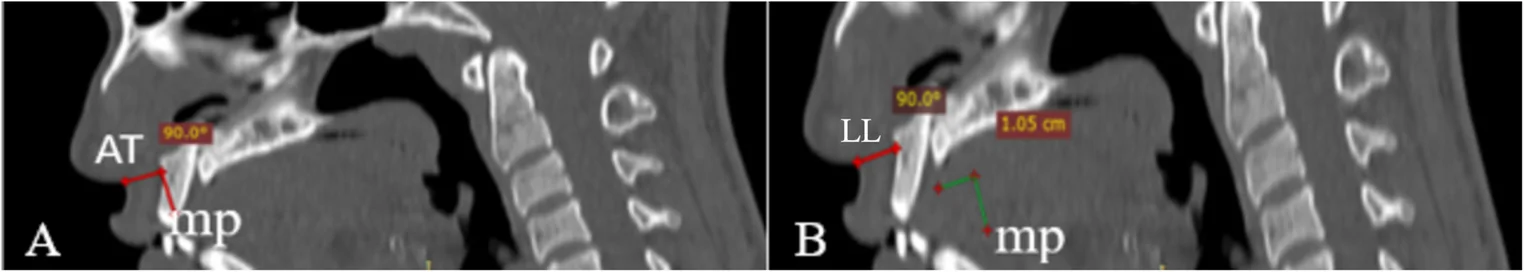
Use of RadiAnt in FSTT measurements. (**a**) Angle tool (AT, red colour) placed at 90°. (**b**) Linear line (LL, in red colour) placed for measurement

After identifying the desired facial soft tissue thickness point, the angle tool had to be manoeuvred to 90° from bone to skin, for example, at the mp (mid-philtrum), Fig. 3A. The manipulation was time-consuming, as it was impossible to pre-program the angle tool to take each measurement at 90°. Secondarily, after placing the angle tool (AT) at 90°, a measuring tool (LL) was then placed directly over the angle tool to estimate the representative distance from bone to skin surface, Fig. 3B. The angle tool could then be deleted, leaving only the linear line. A total of 3101 manual measurements were taken at these FSTT points.

## Results

**Fig. 4:**
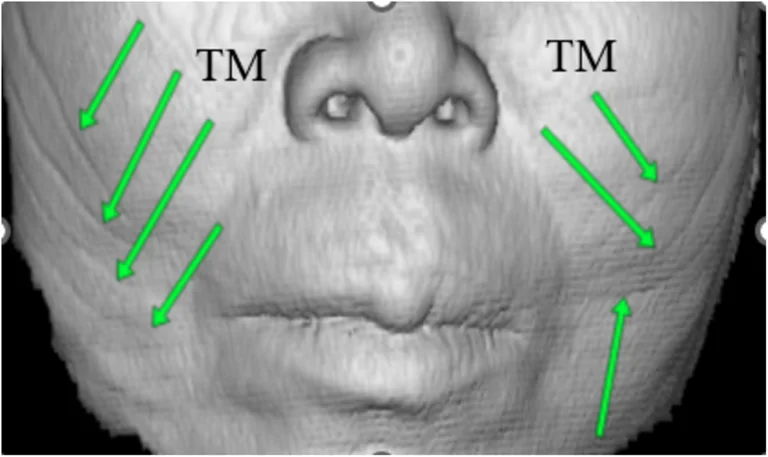
A subject with ethnic tribal marks (TM) on both cheeks, defined by the end of the green arrowhead

Thirty-five computed tomography scans were analysed. Three were discarded because one was distorted, another had a significant lesion (swelling) in the parotid region and another because of tribal marks (TM) as illustrated in Fig. 4.

**Fig. 5:**
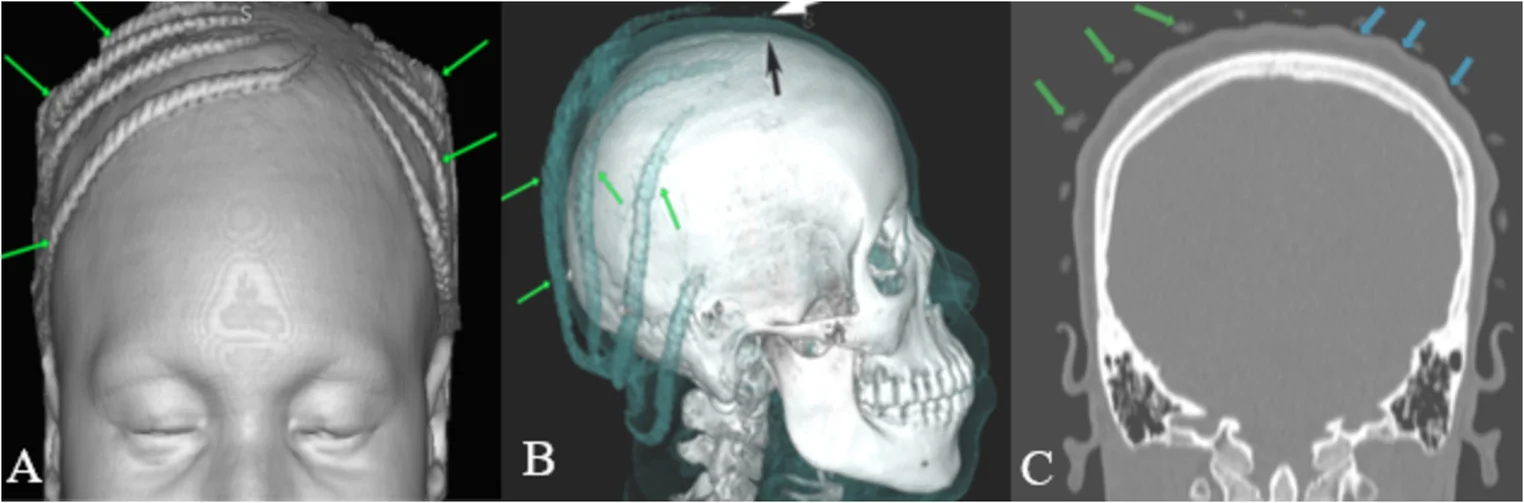
Hair tie woven style **(a)** in 3D reformat, **(b)** in bony window, and **(c)** pulling effect in coronal view. The end of the arrowhead defines the point of interest

The 3-dimensional reformat scans of subjects showed a hair tie woven style, Figs. 5 A and 5B (defined by the end of the green arrowhead). When examined further on coronal views, the hair ties had a pulling effect on the scalp, with C-shaped shallow trenches (indicated by the end of the blue arrowhead) and crests of hair woven (end of green arrowhead), Fig. 5C.

**Fig. 6:**
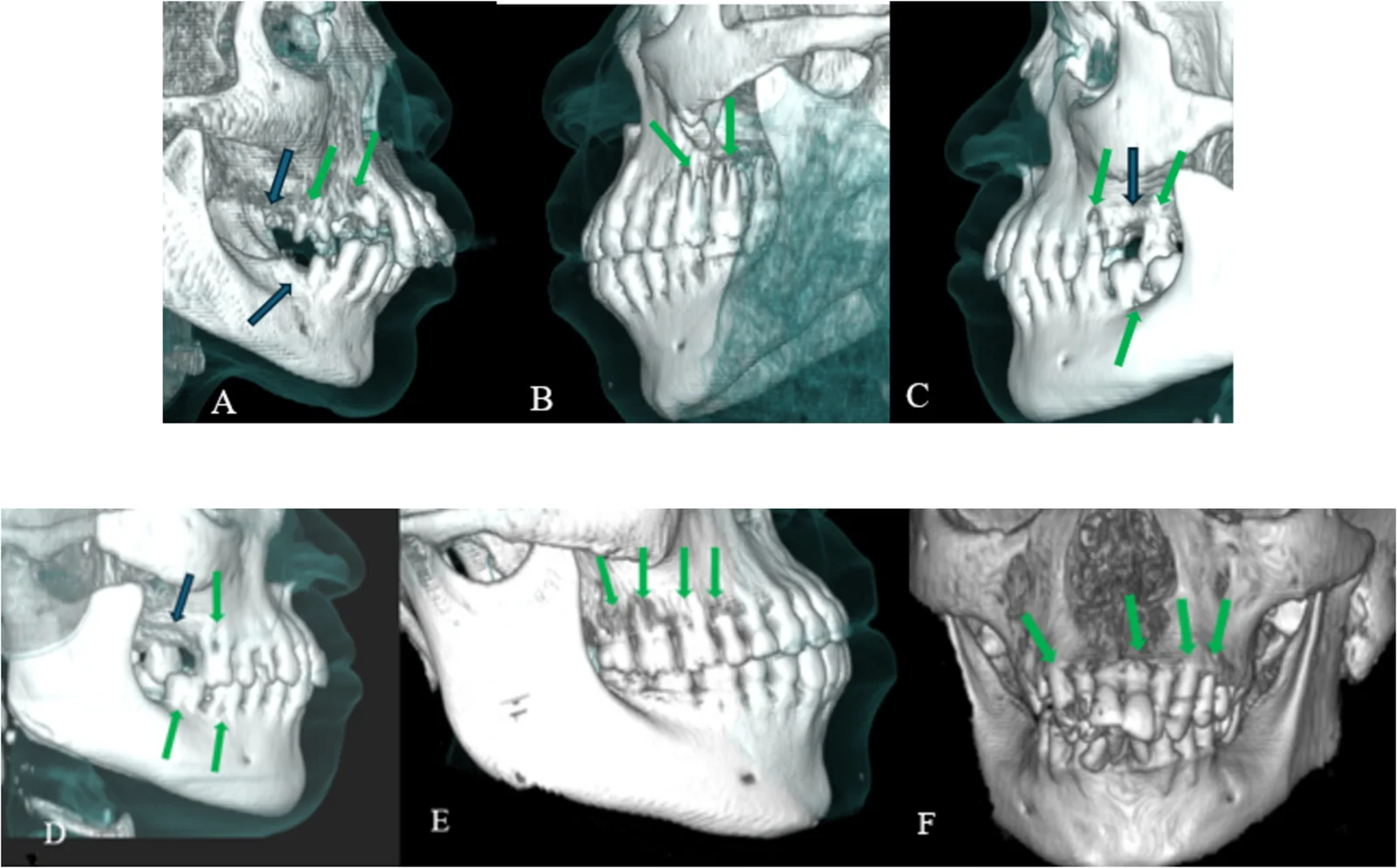
**a** to 6**f** Evidence of advanced periodontal disease and missing teeth as demonstrated in scans. The end of the arrowhead describes points of interest

In addition, the computed tomography bony windows of these subjects showed features of advanced periodontal disease, as illustrated in Figs. 6 A to 6 F and defined by the end of green arrowheads. Also, subjects showed missing teeth as defined by the end of the blue arrowhead. These pathology could affect the thickness of the mid-masseter and other muscles of mastication [43].

Statistical evaluations of these facial soft tissue thickness data were performed using SPSS (IBM Corporation, 1989, 2023) software. To lessen intra- and inter-observer errors, the measurements of the first ten subjects were completed thrice by the same researcher. The p-values of less than 0.05 showed reproducibility of good accuracy in measurements. All subjects were classified based on ethnicity and age brackets, Table 4, and by age, Appendix Table (Appendix 1). The Hausas are the majority subgroup.

**Table 4:** The number of female subjects by ethnic types

Age	Hausa	Fulani	Yoruba	Dakarkari	Total
18–32	10	1	-	-	11
33–47	4	-	1	-	5
48–62	7	2	-	-	9
63–77	2	1	-	1	4
78–92	-	2	-	-	2
93–100	1	-	-	-	1
Total	24	6	1	1	32

The younger age brackets were the most populated. After 63 years, there is a reduction in the number of subjects; presumably a reflection of the reduced life span of 54.95 years for this population [49].

The descriptive statistics for mean, minimum (min), maximum (max), range, standard deviation (STD), and counts of all measured midline facial soft tissue thickness points were calculated for the combined Nigerian female ethnicities (CNFE); see Table 5 below.

These values will be relevant in forensic facial reconstruction of deceased Nigerian female adults aged 18 years and above, especially if the age, ethnicity and body mass index are not known. This type of average values with no age sub-classification [24, 28, 39, 41] nor BMI [32, 37, 39] or weight classification has been published in various works. This dataset is similar to the data for the Nigerian adult male (Table 5).

**Table 5:** Mean values of midline FSTT points for the CNFE (mm)

FSTT point	Mean	Min	Max	Range	STD	Count	Mean midline values for Nigerian males
Vertex	5.54	1.18	9.96	8.78	2.51	29	6.39
Supraglabella	3.80	1.99	5.28	3.29	0.96	32	4.56
Glabella	5.39	3.17	8.49	5.32	1.41	32	5.46
Nasion	5.33	3.31	8.93	5.62	1.51	32	6.09
Mid-Nasal	3.25	1.84	6.06	4.22	0.97	32	3.97
Rhinion	2.59	1.71	4.24	2.53	0.59	32	3.35
Midphiltrum	10.80	6.96	14.7	7.74	1.93	32	12.64
Upper lip border	8.65	5.6	12	6.4	1.46	32	10.17
Lower lip border	10.45	5.39	14.5	9.11	2.76	31	10.79
Labiomental	11.79	6.42	17.2	10.78	3.66	30	12.4
Mental eminence	10.81	6.36	16.0	9.64	3.08	31	10.36
Menton	5.70	2.66	11.3	8.64	2.41	29	6.29

The descriptive statistics for both right and left sides of the face were also calculated (Table 6). The lowest facial soft tissue thickness is at the mlo point, a value of 3.25. The highest value for the Nigerian female is 22.20 at the lm point. The values for the right side of the face were compared with the left, Table 6 (comparison Table); there is marginally more tissue on the left side, which is consistent at all points. Bilateral differences (net differences between the right and left sides) for the Nigerian female were compared with the published data for the Nigerian male [43] (Table 6, comparison Table). 

Descriptive statistics of facial soft tissue thickness for the Hausa female (HF) age range in years of 18 to 32 were estimated, Table 7. 

The Nigerian adult male marginally displayed more soft tissue at some points (fe, lg, ln, sc, ic, mm) except at the lateral sides of the face (lm, sm2, lo, io, za, im2), where females show more soft tissue thickness, Table 8. These differences are also illustrated in (Appendix 2 and 3).

**Table 6:** Descriptive data of bilateral FSTT for the CNFE

Right of CNFE							Left of CNFE							Comparison Table of CNFE CNME			
FSTT points	Mean	Min	Max	Range	SD	Count	Mean	Min	Max	Range	SD	Count	D of CNFE lt - rt	*D of CNFE lt - rt	CNFE lt > rt (%) RD	D of CNME lt - rt	CNME lt > rt RD (%)
Frontal eminence	4.36	1.6	7.69	6.09	1.40	32	4.47	2.27	7.8	5.53	1.34	32	0.11	0.08	2.43	0.39	8.18
Supraorbital	5.43	2.26	8.08	5.82	1.48	32	5.58	2.38	8.73	6.35	1.52	32	0.15	0.10	2.73	0.32	5.58
Infraorbital	10.41	5.77	15.4	9.63	2.88	32	10.50	6.53	15	8.47	2.71	32	0.09	0.03	0.85	0.15	1.59
Supraglenoid	12.24	7.48	18.0	10.52	3.15	32	12.98	7.71	20	12.29	3.34	32	0.74	0.23	6.08	0.93	7.41
Zygomatic arch	6.76	3.21	13.6	10.39	2.62	32	7.39	3.33	13.5	10.17	2.86	32	0.63	0.23	9.28	0.3	4.79
Lateral Orbit	7.65	3.61	12.5	8.89	2.51	32	8.13	3.87	13.4	9.53	2.50	32	0.48	0.19	6.23	0.32	5.28
Lateral Glabella	4.44	1.94	6.87	4.93	1.52	32	4.55	1.81	7.13	5.32	1.59	32	0.11	0.07	2.42	0.13	2.64
Lateral nasal	6.95	3.93	11.8	7.87	1.90	32	7.40	4.32	14.8	10.48	2.19	32	0.45	0.22	6.43	0.32	4.31
lateral maxilla	22.20	14.8	28.9	14.1	5.18	31	23.60	16.0	30.1	14.1	5.29	31	1.40	0.27	6.32	0.93	4.63
Lateral nostril	9.11	6.81	12.0	5.19	2.26	31	9.39	7.01	12.2	5.19	2.31	31	0.28	0.12	3.05	0.5	5.36
Supracanine	7.59	5.15	10.6	5.45	1.97	31	7.98	5.36	11.8	6.44	2.11	31	0.39	0.19	5.09	0.31	3.52
Infra-canine	7.58	2.64	11.6	8.96	3.04	28	7.84	3.2	11.6	8.4	3.13	28	0.26	0.08	3.47	0.39	4.63
Mid-lateral orbit	3.25	1.3	5.45	4.15	1.22	31	3.50	1.33	5.8	4.47	1.27	31	0.25	0.20	7.58	0.19	5.26
Supra-M2	21.64	11.8	34.9	23.1	6.05	31	23.05	14.7	36.4	21.7	7.25	31	1.41	0.21	6.64	0.81	4.06
Infra-M2	17.86	11.2	30.1	18.9	5.13	31	19.02	13.2	32,7	19.5	5.92	31	1.16	0.21	6.47	0.62	3.58
Mid-masseter	20.22	11.7	32.0	20.3	5.63	31	20.98	14.5	28.1	13.6	5.28	31	0.76	0.14	3.75	0.33	1.53
Gonion	16.75	5.73	26.2	20.47	7.00	29	17.53	6.6	28.3	21.7	7.55	29	0.78	0.11	4.64	1.04	6.28
Occlusal line	19.61	9.17	29.0	19.83	7.57	29	20.62	10.6	29.3	18.7	8.07	29	1.10	0.13	5.15	1.09	5.59
Mid-mandible	11.88	4.46	22.0	17.54	5.38	28	12.64	5.01	22.3	17.29	5.83	28	0.76	0.14	6.43	0.58	4.95

CNFE= combined Nigerian female ethnicities, CNME= combined Nigerian male ethnicities. D= differences in mean, *D= cohen’s D, RD= relative difference, rt= right, lt= left

**Table 7:** FSTT values for HF (18-32), midline and right face (mm)

18–32						
FSTT point (midline)	Mean	Min	Max	Range	SD	Count
Vertex	7.50	6.22	9.96	3.74	3.36	8
Supraglabella	3.54	2.28	4.91	2.63	1.06	10
Glabella	4.76	3.35	6.56	3.21	1.14	10
Nasion	4.87	3.31	6.79	3.48	1.14	10
Mid-Nasal	3.55	2.36	5.86	3.50	0.95	10
Rhinion	2.56	2.09	3.53	1.44	0.44	10
Midphiltrum	11.08	7.40	14.30	6.90	2.30	10
Upper lip border	9.32	6.93	12.00	5.07	1.72	10
Lower lip border	9.67	8.05	13.80	5.75	1.83	10
Labiomental point	11.45	9.60	14.90	5.30	1.65	10
Mental eminence	10.76	6.36	14.30	7.94	2.13	10
Menton	4.97	2.66	6.85	4.19	2.45	8
18–32						
FSTT point (right face)	Mean	Min	Max	Range	SD	Count
Frontal eminence	4.53	3.51	6.16	2.65	1.00	10
Supraorbital	5.80	4.06	8.08	4.02	1.22	10
Infraorbital	8.86	5.77	13.80	8.03	2.50	10
Supraglenoid	11.69	7.48	15.40	7.92	2.63	10
Zygomatic arch	6.92	4.17	11.20	7.03	2.05	10
Lateral Orbit	7.42	5.05	12.50	7.45	2.28	10
Lateral Glabella	4.65	1.94	6.69	4.75	1.74	10
Lateral nasal	6.10	3.93	11.80	7.87	2.41	10
lateral maxilla	20.28	14.80	27.80	13.00	3.86	10
Lateral nostril	9.46	7.05	12.00	4.95	1.63	10
supracanine	8.34	5.92	10.60	4.68	1.77	10
Infra-canine	7.86	4.92	11.60	6.68	2.11	10
Mid-lateral orbit	3.48	2.24	5.26	3.02	0.99	10
Supra-M2	21.34	15.30	28.00	12.70	4.71	10
Infra-M2	18.40	11.20	26.40	15.20	4.85	10
Mid-masseter	19.34	15.10	24.50	9.40	3.49	10
Gonion	15.15	5.73	22.70	16.97	5.82	10
Occlusal line	17.64	9.17	24.90	15.73	6.06	10
Mid-mandible	10.58	4.46	18.30	13.84	5.30	9

Hausa female = (HF), FSTT= facial soft tissue thickness

**Table 8:** Differences between the CNFE and CNME FSTT data (mm)

FSTT	CNFE	CNME	CNME (18–32)	HF° (18–32)	Comparison Table	
					CNME – CNFE (Both these are the combined adult values)	CNME* (18–32) – HF° (18–32)
Vertex	5.54	6.39	7.27	7.5	0.85	−0.23
Supraglabella	3.80	4.56	4.85	3.54	0.76	1.31
Glabella	5.39	5.46	6.06	4.76	0.07	1.3
Nasion	5.33	6.09	6.63	4.87	0.76	1.76*
Mid-nasal	3.25	3.97	4.37	3.55	0.72	0.82
Rhinion	2.59	3.35	3.31	2.56	0.76	0.75
Midphiltrum	10.80	12.64	13.31	11.08	1.84*	2.23*
Upper lip border	8.65	10.17	10.92	9.32	1.52*	1.6*
Lower lip border	10.45	10.79	10.44	9.67	0.34	0.77
Labiomental point	11.79	12.4	11.91	11.45	0.61	0.46
Mental eminence	10.81	10.36	10.11	10.76	−0.45	−0.65
Menton	5.70	6.29	6.33	4.97	0.59	1.36
Frontal eminence	4.36	4.77	4.46	4.53	0.41	−0.07
Supraorbital	5.43	5.73	6.14	5.8	0.3	0.34
Infraorbital	10.41	9.44	9.87	8.86	−0.97	1.01
Supraglenoid	12.24	12.55	12.58	11.69	0.31	0.89
Zygomatic arch	6.76	6.26	6.27	6.92	−0.5	−0.65
Lateral Orbit	7.65	6.06	6.01	7.42	−1.59*	−1.41
Lateral Glabella	4.44	4.92	5.50	4.65	0.48	0.85
Lateral nasal	6.95	7.43	8.41	6.1	0.48	2.31*
lateral maxilla	22.20	20.09	20.23	20.28	−2.11*	−0.05
Lateral nostril	9.11	9.33	9.77	9.46	0.22	0.31
supracanine	7.59	8.80	9.54	8.34	1.21	1.2
Infra-canine	7.58	8.42	8.91	7.86	0.84	1.05
Mid-lateral orbit	3.25	3.61	3.94	3.48	0.36	0.46
Supra-M2	21.64	19.93	21.24	21.34	−1.71*	−0.1
Infra-M2	17.86	17.34	18.50	18.4	−0.52	0.1
Mid-masseter	20.22	21.54	22.49	19.34	1.32	3.15*
Gonion	16.75	16.57	16.45	15.15	−0.18	1.3
Occlusal line	19.61	19.51	20.63	17.64	−0.1	2.99*
Mid-mandible	11.88	11.71	11.27	10.58	−0.17	0.69

This type of age sub-classification has been reported in other FSTT studies [2, 50]. The Hausa female (HF) (18 to 32) midline and right-side soft tissue thicknesses were compared with published data for the Nigerian male population of the same young adults (18 to 32 years) (Table 8). As with the older adult, the young adult male data show slight increases in values, but again not at the lateral side of the face, where there is a marginally more soft tissue at the za and lo in females (Table 8, comparative Table).

## Discussions

Facial soft tissue thickness (FSTT) is an index used in forensic facial reconstruction (FFR), and values vary between different populations [1, 2, 5, 17], [40, 51, 52]. Approximated population values of FSTT are commonly used in FFR [52]. It has been postulated that the differences in FSTT values between different populations are due to variations in the type of imaging modality, such as magnetic resonance imaging, computed tomography (CT), ultrasound, and cone beam computed tomography [52] or slight differences in bone morphology between populations. Computed tomography images are very reliable at demonstrating craniofacial bone and soft tissues [24, 43, 52]. In this study, the computed tomography machine was the same as that used in the previous FSTT publication on the combined Nigerian male adult (CNME) data [43].

In comparing the findings of this study with any other published population data, similar to the CNME study, acceptable point variation should be at least a value of 1.5 mm. Previous studies have stated that a difference of less than 1 mm is an insignificant benchmark [43, 52]. In comparison to other studies (Table 1), this study has included an average number of subjects. When compared to previous studies in Africa, a greater number of FSTT points have been used (Table 9), and this should increase coverage of the facial contours. Furthermore, none of the African studies measured or compared the right and left sides of the face in the Nigerian female adult.

**Table 9:** List of FSTT studies in Africa

Population studied	Image type/number of FSTT points	M/F	Number of patients, age range (years)	Study name/year (abbreviation)
Zulus (South Africa)	Lateral and oblique cephalometric + ultrasound scan/54	M	55, 20 to 35	Aulsebrook et al., 1995 (A°) [26]
Mixed population, White and Black (Cape areas, South Africa)	Computed tomography scan at 5 mm slides whilst using about 20 slides per patient/32	M/F	32 (16 male, 16 female), 12 to 71	Phillip and Smith, 1996 (P & S) [40]
Black Population (Gauteng region, South Africa)	The computed tomography thickness of the slides is not stated. Measurements were from 28 FSTT points midline, 10 of which were midline/28	F	154 CT scans, 18 to 35	Cavanagh and Steyn, 2011 (C & S) [39]
Sudanese ethnic Group	Plane Cephalogram/20	M/F	233, 18 to 35	Hamid and Abuaffan, 2016 (H & A) [35]
Egyptian	Ultrasound/17	M/F	204, 20–35	El-Mehallawi and Soliman [37]
Egyptian	Cone-beam computed tomography/18 (10 midline, 8 bilaterally)	M/F	20 (M = 10, F = 10)/17–33	Shehata et al., 2023 [41]
Nigerian	Computed tomography/50	M	55, 18–100	Adegbite et al. [43]
Nigerian	Computed tomography/50	F	32, 18 to 96	This study

The RadiAnt software used for estimating the distance from bone to skin at each FSTT point produces a good 3-D reformat of the images, thereby allowing for the elimination of some scans and optimising measurements and results.

### Analysis and comparison of the midline data

**Fig. 7:**
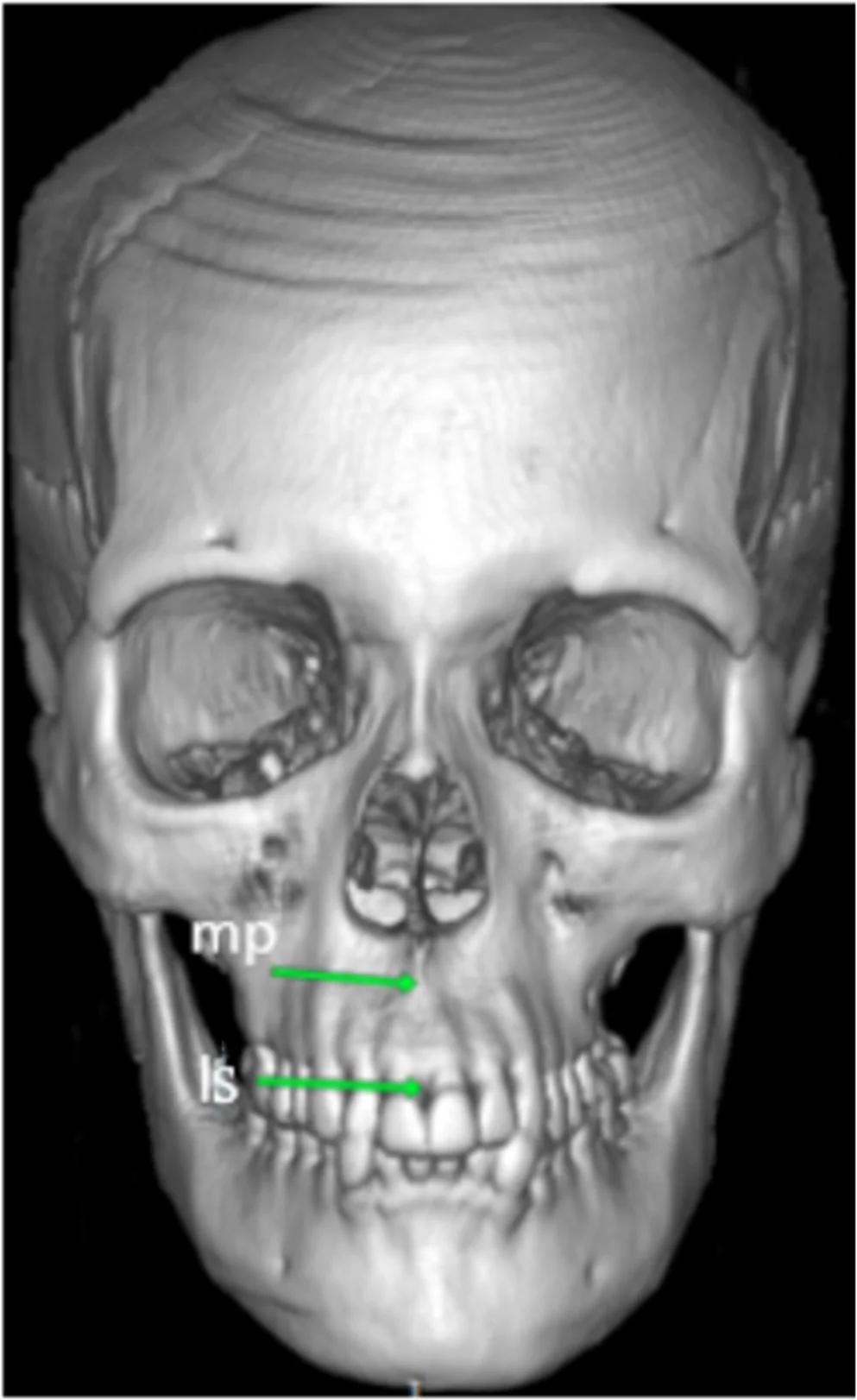
Points with more soft tissue in Nigerian males (defined by the end of the green arrowhead)

The soft tissue thickness point with the lowest value for the combined Nigerian female adult (CNFE) is at the rh point with a value of 2.59 mm; this is lower than the 3.35 mm value for the Nigerian male adult (CNME). Studies, including the (CNME), have reported this as the point with the lowest value [38, 42, 43]. The FSTT with the highest value is at the lm point. This is similar to the CNME, which had its highest and similar values at the mp and lm points. As illustrated in Table 5; Fig. 7, CNME displayed marginally higher values at most midline points and with more significant differences at the mp (1.84) and ls (1.52), respectively; except for an insignificantly higher CNFE value at the me (10.81 mm) as compared to the CNME (10.36 mm).

Despite the hair-woven pulling pattern in the CNFE, Fig. 5, the CNME displaced more value at the vertex (5.54 mm vs. 6.39 mm). The CNME report shows slightly more soft tissue beneath the chin (mn) point than the CNFE; this is contrary to the previous report by Wilkinson [1] that females are likely to have greater volume of soft tissue at this point. One previous study has shown that, based on the same body mass index (BMI), females tend to have more facial soft tissue thickness than males [53]. It should be noted that detailed BMI has not been investigated in this study. 

### Analysis and comparison of bilateral data

The facial soft tissue thickness (FSTT) data for both sides of the face were analysed and compared with the CNME data and other populations’ published data. Like the CNME, the CNFE’s lowest FSTT value was at the mid-lateral orbit (mlo). CNFE’s highest FSTT value was at the lateral maxilla (lm) point; this is dissimilar to the CNME, which had its highest value at the mid-masseter (mm) point.

**Fig. 8:**
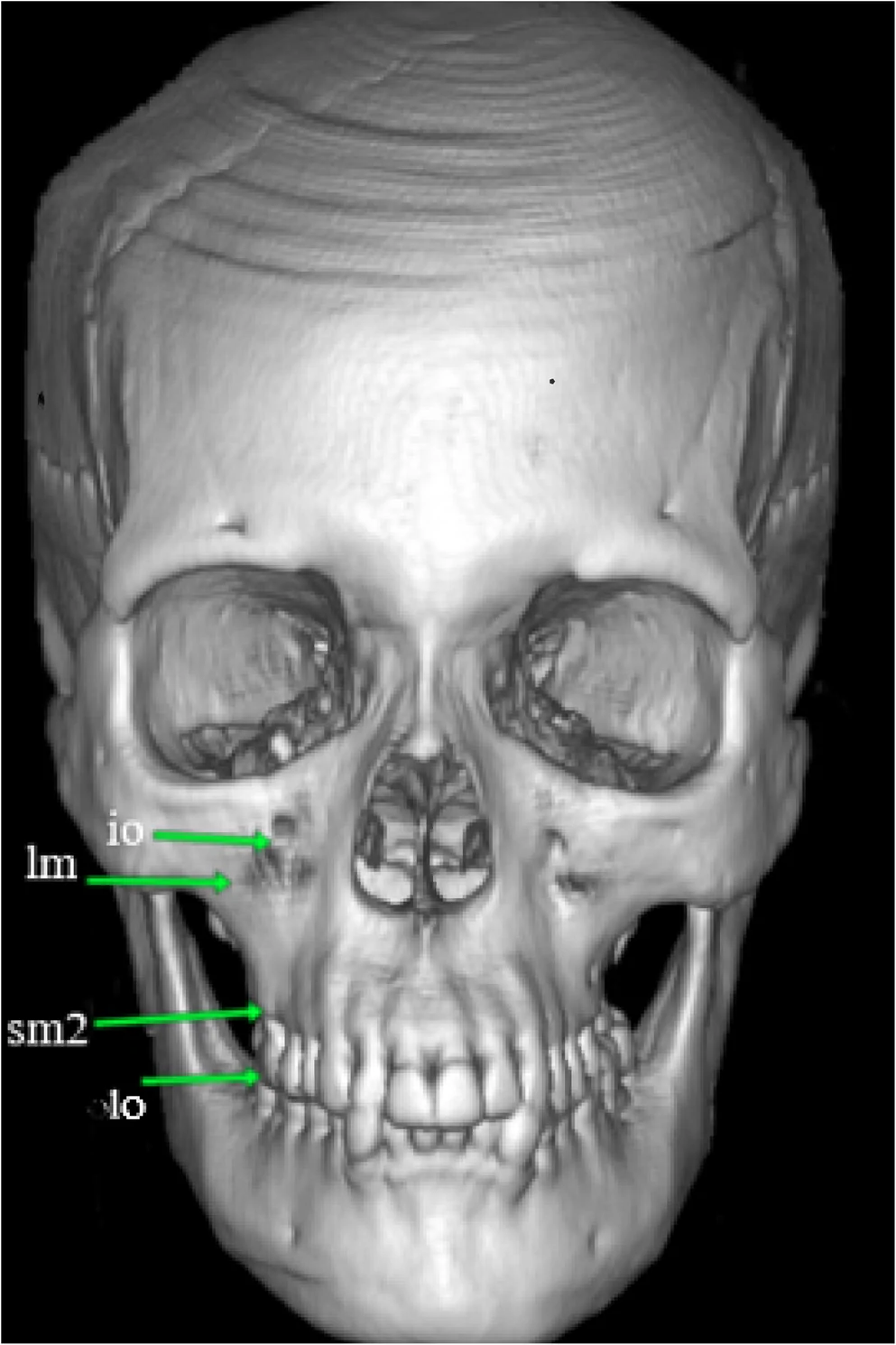
Points with more soft tissue in Nigerian females (defined by the end of the green arrowhead)

Comparing the Nigerian female and male represented as mean difference (comparison column) in Table 6 shows that males have marginally more soft tissue at more FSTT points, except at the lateral points of the face where females shows marginally more soft tissues, including at the lm (22.20 vs. 20.09), sm2 (21.64 vs. 19.93), lo (7.65 vs. 6.06), io (10.41 vs. 9.44), and za (6.76 vs. 6.26), respectively (Table 8; Fig. 8). The increased values at the zygomatic arch, lateral orbit, lateral maxilla, and supra-M2 in the CNFE is consistent with previous studies, including studies on Japanese, white Europeans, black and white Americans [1, 21, 31, 38].

Similar to the male, the differences between the right and left faces of the Nigerian female consistently reveal (marginally) more soft tissues on the left side at all FSTT points (Table 6, comparative Table), also seen in Cohen’s D difference. The highest differences between left and right sides of the face are at the supra-M2, lateral maxilla, and the infra-M2 points with differential values of 1.41, 1.4, and 1.16 mm, respectively.

The Nigerian adult female FSTT points with the least relative percentage difference were at the infraorbital (0.85%), lateral glabella (2.42%), and frontal eminence (2.43%) regions, Table 6. The highest relative percentage difference was at the zygomatic arch (9.28%), the mid-lateral orbit (7.58%) and the supra-M2 (6.64%). This is different to the Nigerian males, the FSTT point with the least relative difference is at the mid-masseter (1.53%), and the highest is at the frontal eminence (8.18%), Table 6.

These Nigerian adult female (CNFE) bilateral data were compared with other population studies that have compared the right and left sides of the face (Table 10). At least for this Nigerian adult female and male, these differences were not due to the head position because the same machine was used and each subject was scanned in a supine position with the head centred by equal support to both the left and right sides of the face. Those that were not well-centred were rejected from the dataset as the measurement could not be standardised.

**Table 10:** Comparing data for bilateral FSTT of CNFE with other populations (mm)

FSTT points	Study name						
	Lt Rt						
	CNFE	R & C (female) [38]	R & C (male) [38]	De Geoff et al., [33]	Hwang et al., [28]	Toneva et al., (female) [51]	Toneva et al., (male) [51]
Frontal eminence	4.47 4.36	8.00 8.00	8.25 8.75	4.1 4.3	5.9 6.0	- -	- -
Supraorbital	5.58 5.43	4.50 4.50	4.75 4.75	5.6 5.4	6.7 6.8	6.73 6.96	7.59 7.79
Infraorbital	10.50 10.41	8.50 8.25	7.50 7.75	9.5 9.4	7.6 7.5	5.11 5.28	5.40 5.27
Supraglenoid	12.98 12.24	12.00 12.25	11.75 11.75	9.9 9.5	12.2 12.1	- -	- -
Zygomatic arch	7.39 6.76	9.25 9.00	8.75 8.50	6.5 6.7	8.5 8.6	8.33 8.07	7.29 6.98
Lateral Orbit	8.13 7.65	14.25* 12.75	13.00 13.00	8.9 9.4	9.4 9.3	8.75 8.67	7.10 7.38
Lateral Glabella	4.55 4.44	- -	- -	5.7 5.9	8.8 8.6	- -	- -
Lateral nasal	7.40 6.95	- -	- -	3.8 3.7	6.9 7.0	- -	- -
lateral maxilla	23.60 22.20	- -	- -	17.5 18.2	18.3 18.3	- -	- -
Lateral nostril	9.39 9.11	- -	- -	9.7 9.5	13.5 13.7	- -	- -
Supracanine	7.98 7.59	- -	- -	9.9 9.3	10.8 10.9	- -	- -
Infra-canine	7.84 7.58	- -	- -	10.0 10.5	11.7 11.9	- -	- -
Mid-lateral orbit	3.50 3.25	- -	- -	4.8 4.8	5.0 5.0	3.26 3.44	3.18 3.48
Supra-M2	23.05 21.64	20.75 21.25	22.25 22.00	26.6 26.8	27.9 27.8	- -	- -
Infra-M2	19.02 17.86	16.75 17.25	15.75 16.50	17.9 18.8	20.7 20.6	- -	- -
Mid-masseter	20.98 20.22	- -	- -	16.2 17.8*	18.3 17.8	18.71 19.22	20.21 19.71
Gonion	17.53 16.75	14.25 14.25	14.25 14.75	14.2 15.1	13.0 12.5	13.29 13.10	14.27 13.28
Occlusal line	20.62 19.61	18.25 19.25	19.50 19.00	19.0 19.5	21.9 21.8	21.30 21.33	22.27 22.55
Mid-mandible	12.64 11.88	- -	- -	10.6 12.1*	7.9 7.7	- -	- -

Hwang et al., = (combined male and female averages), - = not documented, * = difference noted, ** = more difference noted

 There are only very few studies which have reported a consistent increase in soft tissue on the left side of the face at all FSTT points; this includes the Sahni et al. on an adult population in northwest India [5]. Coskun reported that the left side of the Greek adult female face was marginally larger than the right side, although in males, the converse was found, as the right side had more thickness than the left, with the most difference at the zygomaxillary [34].

Table 10 reveals that the differences at FSTT points are marginal and not confined to one side of the face but vary at different points on the right and left, also documented by Coskun, Beaini, and other authors [28, 33, 38, 51]. 

In conclusion, the CNFE, like the CNME, had more soft tissue thickness on the left than the right; this is similar to Sahni et al. [5]. These differences are generally marginal and less than 6% [53]. 

### Comparative analysis of the CNFE young adult FSTT with other populations of similar age 

**Fig. 9:**
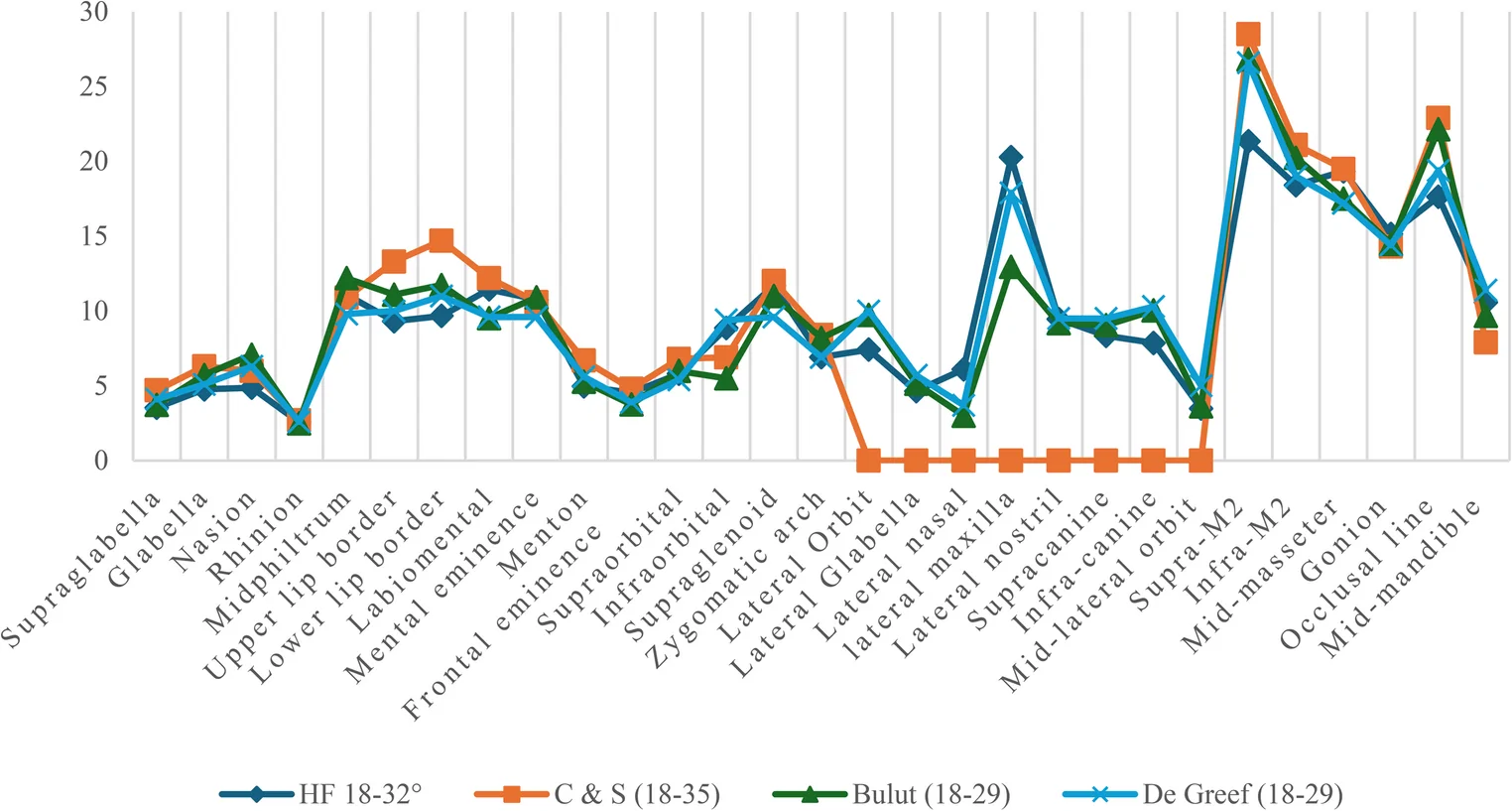
Compares the (HF°)18–32 with other populations

The young Hausa female 18 to 32 years range (represented as HF°) was compared with the young Nigerian male adults (CNME of age 18 to 32 years) and with other populations (Tables 8 and 11). The CNME 18 to 32 generally displayed greater soft tissue thickness at most FSTT points than the HF° 18–32, but especially at the mm, mp, ol, ln, and ls points with differences of 3.15, 2.23, 2.99, 2.31, and 1.60, respectively (Table 8) and Fig. 9 illustrate the comparison with females in other populations of similar age brackets. The De Geoff female aged 18 to 29 years (Belgium data) represented as De Greef°, when compared with the HF°, shows differences greater than 1.5 mm affecting 10 FSTT points, Table 11.

**Table 11:** Comparison of FSTT for a similar young adult age bracket (mm)

Data Table					Comparison Table		
FSTT points	HF° (18–32)	C & S (18–35) [39]	Bulut° (18–29) [2]	De Greef° (18–29) [33]	HF° - C & S	HF° - Bulut°	HF° - De Greef°
Vertex	7.50	-	-	-	-	-	-
Supraglabella	3.54	4.7	3.73	4.1	−1.16	−0.19	−0.56
Glabella	4.76	6.3	5.72	5.1	−1.54*	−0.96	−0.34
Nasion	4.87	6.0	7.08	6.3	−1.13	−2.21*	−1.43
Rhinion	2.56	2.7	2.49	2.6	−0.14	0.07	−0.04
Midphiltrum	11.08	10.9	12.19	9.8	0.18	−1.11	1.28
Upper lip border	9.32	13.3	11.09	10.0	−3.98**	−1.77*	−0.68
Lower lip border	9.67	14.7	11.73	11.0	−5.03***	−2.06*	−1.33
Labiomental	11.45	12.2	9.52	9.6	−0.75	1.93*	1.85*
Mental eminence	10.76	10.6	10.91	9.6	0.16	−0.15	1.16
Menton	4.97	6.7	5.27	5.6	−1.73*	−0.3	−0.63
Frontal eminence	4.53	4.8	3.75	3.9	−0.27	0.78	0.63
Supraorbital	5.80	6.8	5.99	5.4	−1	−0.19	0.4
Infraorbital	8.86	6.9	5.50	9.4	1.96*	3.36**	−0.54
Supraglenoid	11.69	12.0	11.0	9.6	−0.31	0.69	2.09*
Zygomatic arch	6.92	8.4	8.19	6.9	−1.48	−1.27	− 0.02
Lateral Orbit	7.42	-	9.73	10.0	-	−2.31*	−2.58*
Lateral Glabella	4.65	-	5.14	5.7	-	−0.49	−1.05
Lateral nasal	6.10	-	3.00	3.7	-	3.1**	2.4*
lateral maxilla	20.28	-	12.95	17.9	-	7.33***	2.38*
Lateral nostril	9.46	-	9.18	9.5	-	0.28	−0.04
Supracanine	8.34	-	9.05	9.5	-	−0.71	−1.16
Infra-canine	7.86	-	10.04	10.3	-	−2.18*	−2.44*
Mid-lateral orbit	3.48	-	3.62	5.0	-	−0.14	−1.52*
Supra-M2	21.34	28.5	26.81	26.6	−7.16***	−5.47***	−5.26***
Infra-M2	18.40	21.1	20.24	19.0	−2.7*	−1.84*	−0.6
Mid-masseter	19.34	19.5	17.51	17.2	−0.16	1.83*	2.14*
Gonion	15.15	14.3	14.48	14.4	0.85	0.67	0.75
Occlusal line	17.64	22.9	22.14	19.4	−5.26***	−4.5**	−1.76*
Mid-mandible	10.58	7.9	9.68	11.4	2.68*	0.9	−0.82

**Fig. 10:**
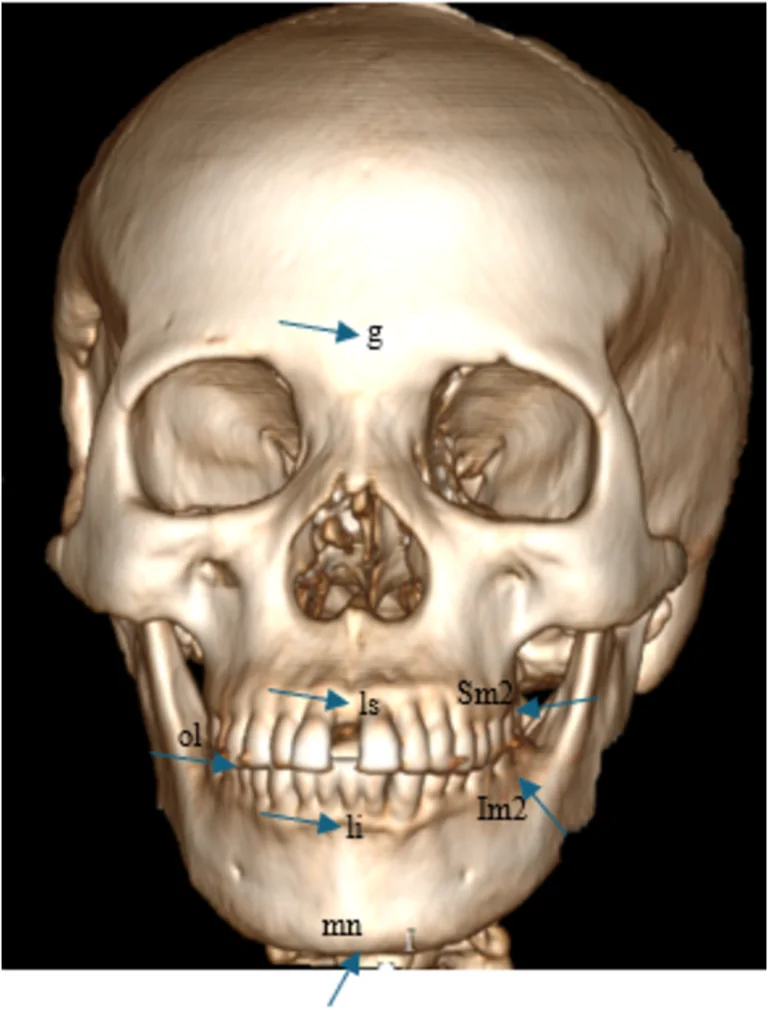
Points with the most differences between HF° and C & S (defined by the end of the blue arrowhead)

The South African females aged 18 to 35 years represented as C & S [39] displayed more thickness at some points than the HF° (18 to 32); these include significant differences at the supra-m2 (7.16), li (5.03), ls (3.98), infra-m2 (2.7), io (1.96), mn (1.73) and g (1.54), Fig. 10.

**Fig. 11:**
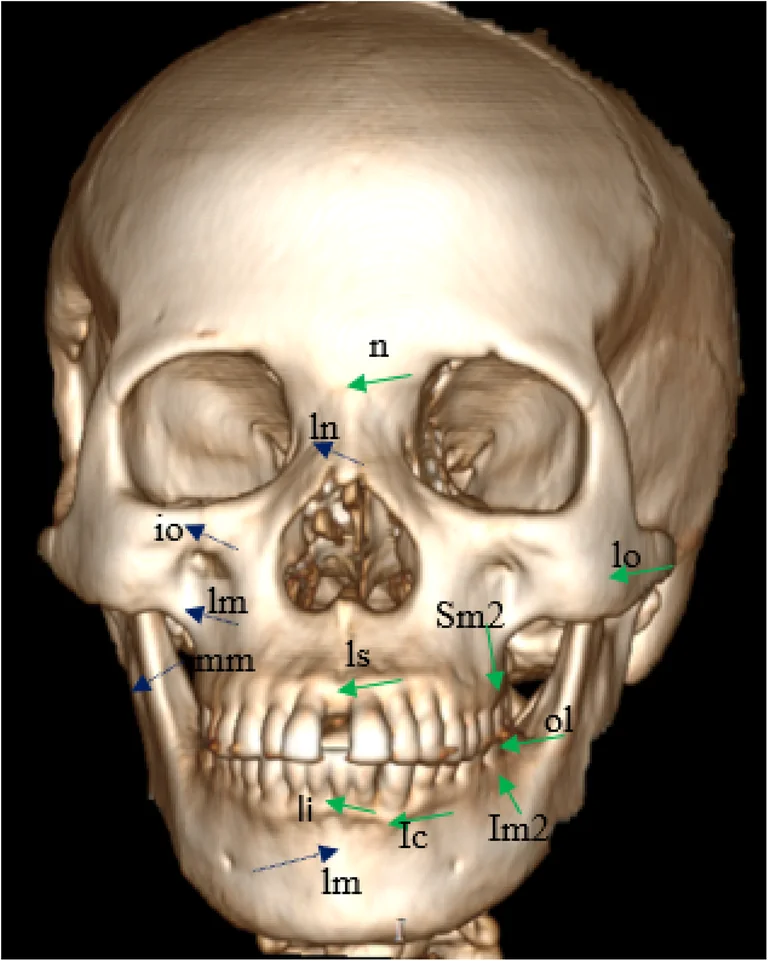
FSTT points with the most differences between HF° and Bulut et al 18-29° (defined by the end of the green and blue arrowhead)

Bulut et al. on subjects aged 18 to 29 years represented as Bulut° (Turkey data) [2] also show more soft tissue thickness than the HF° at points as indicated on the left face by the end of the green arrowhead, Fig. 11. The HF°, however, demonstrates more thickness at other points as defined by the end of the blue arrowhead on the right face.

Other studies, including Manhein et al. have reported greater soft tissue thickness in black American adult males in the age brackets (19 to 34 and 35 to 45 years). In the 19 to 34 group there are differences of 3.8 mm and 2.1 mm at the mp and mn, respectively [31]. For the 35 to 45 age bracket, this was also prominent at the mp, with the male mean value greater than the female by 2.2 mm. Manhein et al. similarly report more tissue thickness in white American adult males in all age categories (19–34, 35–45, 46–55, > 56) except at the rh and mp in the 46–55 where both sexes had same values and in the > 56 years females, with more FSTT values at the me and mn [31]. This is similar to the Nigerian female, which showed marginally more value at the me point. 

### Comparative analysis of CNFE facial soft tissue with other global adult populations

**Fig. 12:**
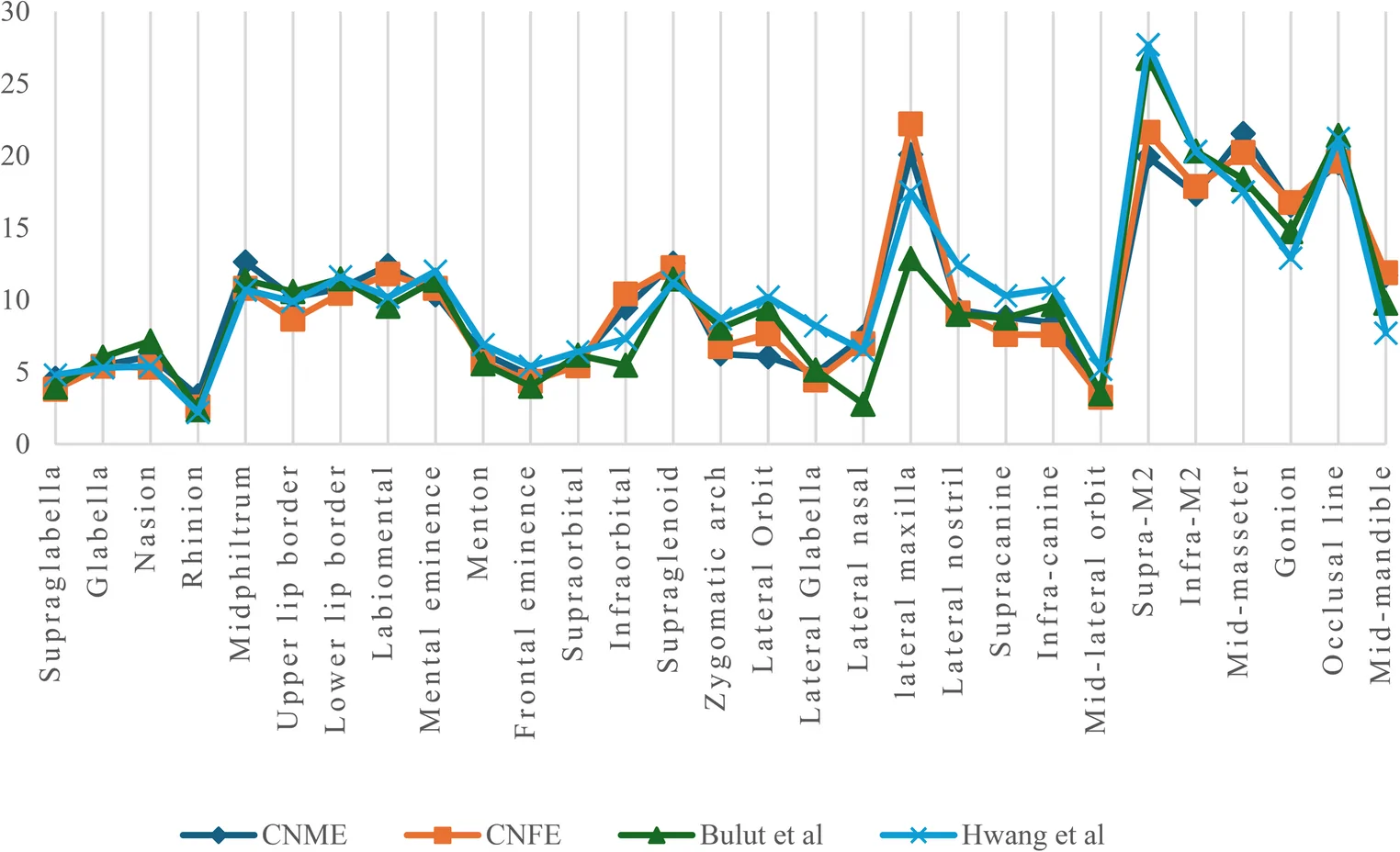
Compares the CNFE FSTT with other population data

The Nigerian adult female (CNFE) right side of the face show soft tissue differences (represented in *) when compared with published values for Turkey and Korea populations, in Table 12; Fig. 12. The CNFE tend to displace more soft tissue thickness at the lateral maxilla with difference of 9.32 and 4.7 more than the data by Bulut et al. and Hwang et al., respectively. Also, CNFE have more thickness at the mid-masseter. However, Bulut et al.‘s data show more tissues at the sm2 (21.64 vs. 26.76) and ol (19.61 vs. 21.41), respectively, as indicated with *.

**Table 12:** Comparative analysis of CNFE FSTT values with other populations (mm)

FSTT points	CNFE	Bulut et al.	Hwang et al.	CNFE -Bulut et al.	CNFE - Hwang et al.
Supraglabella	3.80	3.94	4.8	−0.14	−1
Glabella	5.39	6.03	5.3	−0.64	0.09
Nasion	5.33	7.16	5.4	−1.83*	−0.07
Rhinion	2.59	2.38	2.2	0.21	0.39
Midphiltrum	10.80	11.38	10.7	−0.58	0.1
Upper lip border	8.65	10.58	9.9	−1.93*	−1.25
Lower lip border	10.45	11.47	11.6	−1.02	−1.15
Labiomental	11.79	9.56	10.2	2.23*	1.59*
Mental eminence	10.81	11.38	12.0	−0.57	−1.19
Menton	5.70	5.57	6.9	0.13	−1.2
Frontal eminence	4.36	4.02	5.4	0.34	−1.04
Supraorbital	5.43	6.17	6.4	−0.74	−0.97
Infraorbital	10.41	5.47	7.3	4.94***	3.11**
Supraglenoid	12.24	11.45	11.2	0.79	1.04
Zygomatic arch	6.76	8.06	8.7	−1.3	−1.94*
Lateral Orbit	7.65	9.40	10.2	−1.75*	−2.55**
Lateral Glabella	4.44	5.15	8.2	−0.71	−3.76**
Lateral nasal	6.95	2.77	6.5	4.18***	0.45
lateral maxilla	22.20	12.88	17.5	9.32***	4.7***
Lateral nostril	9.11	9.01	12.4	0.1	−3.29**
Supracanine	7.59	8.75	10.3	−1.16	−2.71*
Infra-canine	7.58	9.67	10.8	−2.09*	−3.22**
Mid-lateral orbit	3.25	3.49	5.2	−0.24	−1.95*
Supra-M2	21.64	26.76	27.7	−5.12***	−6.06***
Infra-M2	17.86	20.33	20.30	−2.47*	−2.44*
Mid-masseter	20.22	18.38	17.50	1.84*	2.72*
Gonion	16.75	14.74	12.9	2.01*	3.85**
Occlusal line	19.61	21.41	21.2	−1.8*	−1.59*
Mid-mandible	11.88	9.78	7.7	2.1*	4.18**

*= indicates a significant increase

### Effect of biological sex on facial soft tissue profiles

The CNFE soft tissue points at the lm and sm2 demonstrate the most sexual dimorphism, displaying mean differences of 2.11 and 1.71, respectively, as illustrated in Fig. 8. The published sexual dimorphism for FSTT points differs between populations. Moritsugui et al. data on Midwest Brazil [30] show less tissue thickness in females than males at all points. These differences are at the ns (6.2 vs. 8.1), mp (13.1 vs. 15.7), ls (10 vs. 13.3), li (9.8 vs. 11.9), supramental (11.9 vs. 12.9), pogonion (mn) (9.9 vs. 11.4), me (7.0 vs. 9.0); these are similar to this Nigerian dataset. It is worthwhile to mention that Moritsugui et al. defined the ls as prosthion. Similarly, Beaini et al. on a multi-ancestral Brazilian population [25] report more soft tissue in males than females, except at the lateral orbit (lo) point, where females marginally displayed more tissue. Similarly, Nigerian females have more tissue at the lo than Nigerian males. Sahni et al., northwest India, reported a sexual dimorphism by using 9 variables and sexed with a male accuracy of 88% and female of 76% [5]. Hwang et al.‘s data on Koreans show more soft tissue values in male adults, particularly at the mp (12.5 vs. 10.7), ls (11.7 vs. 9.9), and li (13.0 vs. 11.6) with differences of 1.8, 1.8, and 1.4, respectively. Hwang’s Korean female adult marginally shows more soft tissues at the lateral face (za, mlo and lo); this is similar to the Nigerian adult female and male. However, other researchers argue that these differences will not define identification [53].

### Limitations and potential sources of error

Computed tomography (CT) and magnetic resonance imaging (MRI) are both reliable in soft tissue imaging [24]. An accurate soft tissue thickness dataset from any of these will improve FFR [54]. This new dataset for the Nigerian adult female has been generated by the use of computed tomography images. However, the standard supine positioning for craniofacial computed tomography and MRI may have introduced error.

This supine positioning will cause a weight (gravity) pulling effect on the lateral soft tissue, thereby reducing the thickness at the mid-sagittal points and increasing the width of the lateral tissues [52, 55, 56]. CT has been shown to yield higher facial soft tissue thickness (FSTT) values than other imaging modalities, including MRI and ultrasound [52, 57, 58]. These may account for some of the differences in this report. The CT machine in this study had a slice thickness of 1.25 mm; this higher thickness may also have inflated values. Higher slice thicknesses may yield lower resolution [59]; perhaps an average of 0.5 to 0.7 mm thickness will give better resolution.

However, live subjects’ CT is more reliable than using cadavers or other imaging types like lateral cephalograms [8, 24, 32, 38]. Furthermore, in comparison to ultrasound, the direct contact of the gel and probe of ultrasound may cause compression of the soft tissues, thereby giving distorted readings [52, 60]. In future studies, a more sensitive CT or MRI will be used to compare with this dataset. The greater number of FSTT points used in this study will allow for greater coverage of the face, and using its own average FSTT will enhance forensic facial reconstruction as documented in previous studies [56]. Also, specialised software packages employed for measurements may affect measurement accuracy [54, 61].

BMI is not well described in this study. However, as documented in other studies, increases in BMI are more likely to increase soft tissue values [1, 2, 15, 33, 36, 45, 57]. In this Nigerian population, normal weight is quite prevalent rather than underweight, overweight, or obesity [46]. A study has shown that based on the same BMI, females tend to have more soft tissue thickness than males [53]. Some studies have also advocated for producing multiple facial reconstructions of the same face, based on different BMI FSTT data [62].

## Conclusion

Facial soft tissue thickness was measured from a computed tomography scan of the face in Nigerian adult females. Measurements were taken at 50 points, including 12 midlines and 19 bilateral (right and left) points. Preliminary facial soft tissue thickness data have now been created for the Nigerian female and young Hausa female adult subgroup, age 18 to 32 years. This preliminary FSTT dataset will suffice for forensic facial reconstruction for the Nigerian adult female. The Hausa female 18 to 32 years data will be more specific for a Hausa adult female if the defined age is within this range.

This study revealed that the left side of the face marginally has more soft tissue thickness than the right side. Similar to those of other populations, the soft tissue thickness of the Nigerian female were mostly less than the corresponding data for the Nigerian adult males This dataset, like the Nigerian male dataset, shows reproducible similarities and differences when compared with other populations, including South African females, Koreans, Belgians, Bulgarians, Brazilians, Greeks and Turkish adults. The biggest limitations of this study are the lack of a body mass index (or weight) classification and the limited number of subjects used. In future works, a larger sample will allow for stratification by weight and age ranges.

## Figures and Tables

**Fig. 13:**
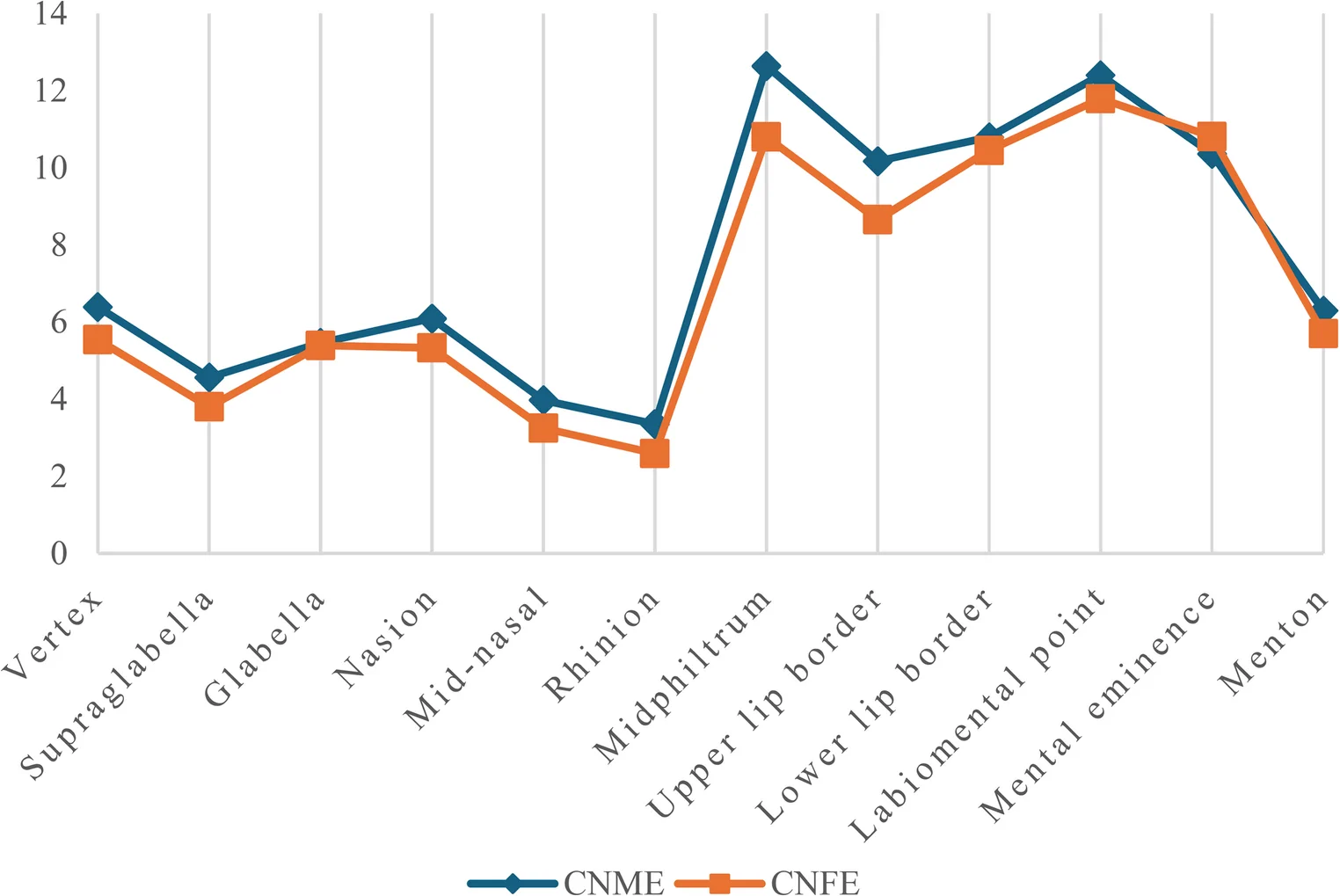
Compares midline mean values of Nigerian male and female (all values in mm)

**Fig. 14:**
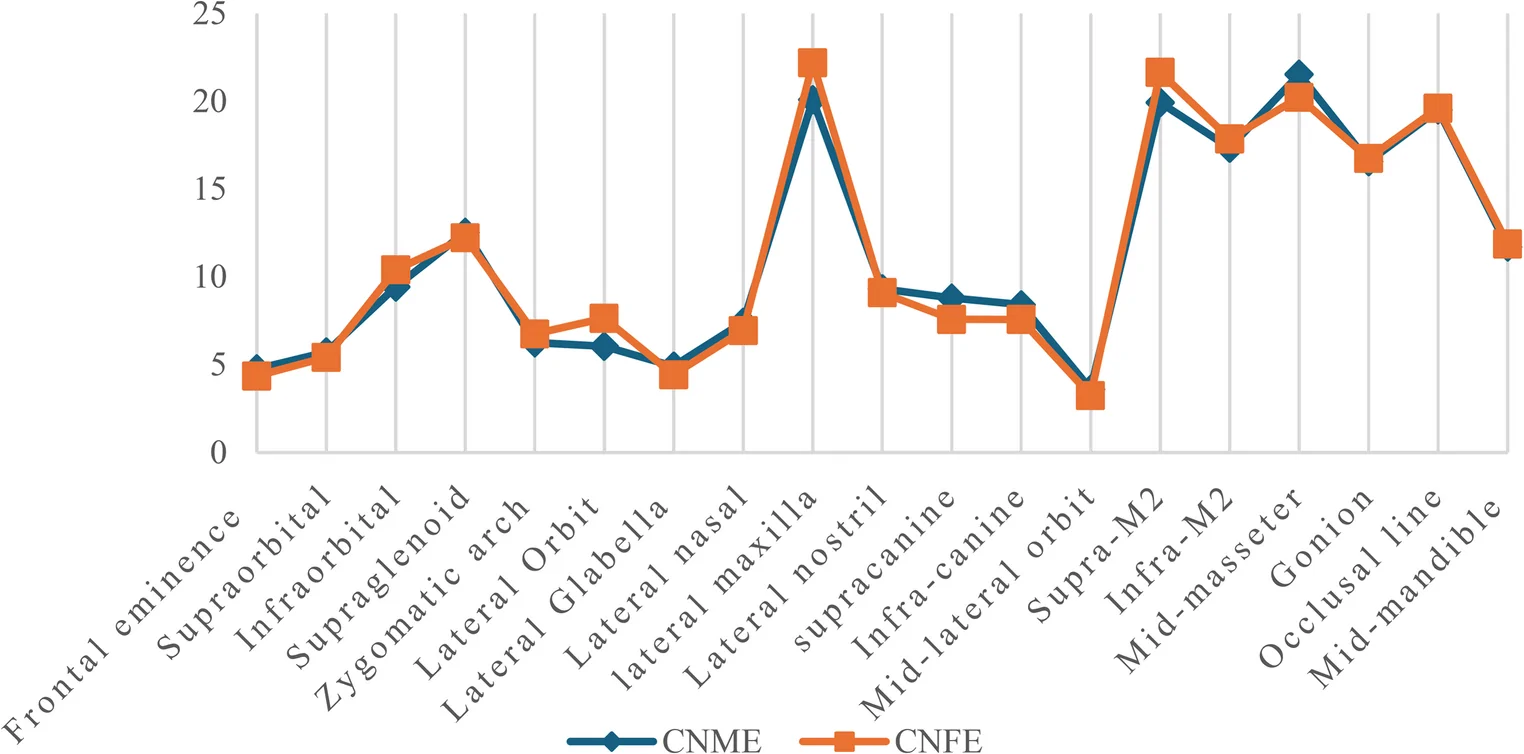
Compares the mean values of the right-face for Nigerian male and female (all values in mm)

## Data Availability

The original raw data are available and can be provided on request. Not published due to the significant proportions of the datasheet.

## Appendix 1

**Table 13:** Appendix Table Ethnic distribution based on age

Age	Hausa	Fulani	Yoruba	Dakarkari	Total
18	2	-	-	-	2
20	2	-	-	-	2
21	1	-	-	-	1
23	1	-	-	-	1
25	2	-	-	-	2
26	1	-	-	-	1
29	1	-	-	-	1
30	-	1	-	-	1
35	1	-	-	-	1
40	1	-	-	-	1
43	-	-	1	-	1
45	2	-	-	-	2
48	1	-	-	-	1
50	2	-	-	-	2
53	1	-	-	-	1
55	2	-	-	-	2
60	1	2	-	-	3
67	1	-	-	1	2
70	1	1	-	-	2
85	-	1	-	-	1
90	-	1	-	-	1
95	1	-	-	-	1
Total	24	6	1	1	32

## References

[1] Wilkinson C (2004) Forensic facial reconstruction. Cambridge University Press

[2] Bulut O, Sipahioglu S, Hekimoglu B (2014) Facial soft tissue thickness database for craniofacial reconstruction in the Turkish adult population. Forensic Sci Int 242:44–61 25023216 10.1016/j.forsciint.2014.06.012

[3] Taylor KT (2000) Forensic art and illustration. CRC Press

[4] Navic P, Palee P, Prapayasatok S, Prasitwattanaseree S, Sinthubua A, Mahakkanukrauh P (2022) The development and testing of Thai facial soft tissue thickness data in three-dimensional computerised forensic facial reconstruction. Med Sci Law 62(2):113–123 34825605 10.1177/00258024211057689

[5] Sahni D, Singh G, Jit I, Singh P et al (2008) Facial soft tissue thickness in northwest Indian adults. Forensic Sci Int 176(2–3):137–146 17997243 10.1016/j.forsciint.2007.07.012

[6] Panenkova´ P, Beˇnuˇs R, Masnicov’a´ S, Obertov´a Z, Grunt J (2012) Facial soft tissue thicknesses of the mid-face for the Slovak population. Forensic Sci Int 220(1–3):293-e1 10.1016/j.forsciint.2012.02.015 22430009

[7] Wilkinson C (2010) Facial reconstruction–anatomical art or artistic anatomy? J Anat 216(2):235–250 20447245 10.1111/j.1469-7580.2009.01182.x PMC2815945

[8] Stephan CN, Simpson EK (2008) Facial soft tissue depths in craniofacial identification (part 1): an analytical review of the published adult data. J Forensic Sci 53(6):1257–1272 18783476 10.1111/j.1556-4029.2008.00852.x

[9] Sahni D, Jit I, Gupta M, Singh P, Suri S, Kaur H (2002) Preliminary study on facial soft tissue thickness by magnetic resonance imaging in northwest Indians. Sahni, daisy (forensic science communications, January 2002.), Research and Technology 4 (1) (2002)

[10] Lebedinskaya GV, Balueva TS, Veselovskaya E (1993) Principles of facial reconstruction In: Iscan and Helmer (eds) Forensic analysis of the skull. Wiley-Liss, Nueva York, pp 183–198

[11] Wilkinson C, Rynn C, Peters H, Taister M, Kau CH, Richmond S (2006) A blind accuracy assessment of computed-modelled forensic facial reconstruction using computed tomography data from live subjects. Forensic Sci Med Pathol 2:179–187 25868696 10.1007/s12024-006-0007-9

[12] Prag J (1997) Making faces using forensic and archaeological evidence

[13] Deng C, Wang D, Chen J, Li K, Yang M, Chen Z, Zhu Z, Yin C, Chen P, Cao D et al (2020) Facial soft tissue thickness in Yangtze River delta Han population: accurate assessment and comparative analysis utilising cone-beam CT. Legal Med 44:101693 32217445 10.1016/j.legalmed.2020.101693

[14] Eftekhari-Moghadam AR, Latifi SM, Nazifi HR, Rezaian J (2020) Influence of sex and body mass index on facial soft tissue thickness measurements in an adult population of southwest of Iran. Surg Radiol Anat 42:627–633 31907581 10.1007/s00276-019-02409-2

[15] Sipahiog˘lu S, Ulubay H, Diren HB (2012) Midline facial soft tissue thickness database of Turkish population: MRI study. Forensic Sci Int 219(1–3):282–e1 10.1016/j.forsciint.2011.11.017 22154437

[16] Snow CC, Gatliff BP, McWilliams KR (1970) Reconstruction of facial features from the skull: an evaluation of its usefulness in forensic anthropology. American Journal of Physical Anthropology 33:221–227. 10.1002/ajpa.1330330207 5473087

[17] Chen Y, Chen Y, Yu Y, Qiang M, Liu D, Fulton T, Chen (2011) Age and sex related measurement of craniofacial soft tissue thickness and nasal profile in the Chinese population. Forensic Sci Int 212(1–3):272–e1 10.1016/j.forsciint.2011.05.027 21715112

[18] Ruiz NAP (2013) Facial soft tissue thickness of Colombian adults. Forensic Sci Int 229(1–3):160–e1 10.1016/j.forsciint.2013.03.017 23587676

[19] Codinha S (2009) Facial soft tissue thicknesses for the Portuguese adult population. Forensic Sci Int 184(1–3):80–e1 10.1016/j.forsciint.2008.11.011 19124207

[20] Guyomarc’h P, Santos F, Dutailly B, Coqueugniot H (2013) Facial soft tissue depths in French adults: variability, specificity and estimation. Forensic Sci Int 231(1–3):411–e1 10.1016/j.forsciint.2013.04.007 23684263

[21] Suzuki H (1948) On the thickness of the soft parts of the Japanese face. J Anthropol Soc Nippon 60:7–11

[22] Rhine JS, Moore CE, Weston J (1982) Facial reproduction: tables of facial tissue thicknesses of American Caucasoids in forensic anthropology. Maxwell Museum of Anthropology, The University of New Mexico

[23] Tedeschi-Oliveira SV, Melani RFH, de Almeida NH, de Paiva LAS (2009) Facial soft tissue thickness of Brazilian adults. Forensic Sci Int 193(1–3):127-e1 10.1016/j.forsciint.2009.09.002 19781879

[24] Domaracki M, Stephan CN (2006) Facial soft tissue thicknesses in Australian adult cadavers. J Forensic Sci 51(1):5–10 16423216 10.1111/j.1556-4029.2005.00009.x

[25] Beaini TL, Miamoto P, Duailibi-Neto EF, Tedeschi-Oliveira SV, Chilvarquer I, Melani RFH (2021) Facial soft tissue depth measurements in cone-beam computed tomography: a study of a Brazilian sample. Legal Med 50:101866 33667933 10.1016/j.legalmed.2021.101866

[26] Aulsebrook W, Becker P, Iscan MY (1996) Facial soft-tissue thicknesses in the adult male Zulu. Forensic Sci Int 79(2):83–102 8698296 10.1016/0379-0738(96)01893-2

[27] Ogawa H (1960) Anatomical study on the Japanese head by the x-ray cephalometry. Shika Gakuho 60:17–34

[28] Hwang H-S, Park M-K, Lee W-J, Cho J-H, Kim B-K, Wilkinson CM (2012) Facial soft tissue thickness database for craniofacial reconstruction in Korean adults. J Forensic Sci 57(6):1442–1447 22621203 10.1111/j.1556-4029.2012.02192.x

[29] Lodha A, Mehta M, Patel M, Menon SK (2016) Facial soft tissue thickness database of Gujarati population for forensic craniofacial reconstruction. Egypt J Forensic Sci 6(2):126–134

[30] Moritsugui DS, Fugiwara FVG, Vassallo FNS, Mazzilli LEN, Beaini TL, Melani RFH (2022) Facial soft tissue thickness in forensic facial reconstruction: impact of regional differences in Brazil. PLoS One 17(7):e0270980 35839226 10.1371/journal.pone.0270980 PMC9286276

[31] Manhein MH, Listi GA, Barsley RE, Musselman R, Barrow NE (2000) Ubelaker, in vivo facial tissue depth measurements for children and adults. J Forensic Sci 45(1):48–60 10641919

[32] Navic P, Sinthubua A, Prasitwattanaseree S, Mahakkanukrauh P (2021) Facial soft tissue thickness for a Thai population using needle puncture technique application for forensic facial reconstruction. Int Med J 28: 552 - 557

[33] De Greef S, Claes P, Vandermeulen D, Mollemans W, Suetens P, Willems G (2006) Large-scale in-vivo Caucasian facial soft tissue thickness database for craniofacial reconstruction. Forensic Sci Int 159:S126–S146 16563680 10.1016/j.forsciint.2006.02.034

[34] Coskun G, Fasoula M, Bontozoglou N (2024) Facial soft tissue depth of a contemporary adult Greek population. International Journal of Legal Medicine 1-19. 10.1007/s00414-024-03305-0 10.1007/s00414-024-03305-0 PMC11490456 39122874

[35] Hamid S, Abuaffan AH (2016) Facial soft tissue thickness in a sample of Sudanese adults with different occlusions. Forensic Sci Int 266:209–214 27314547 10.1016/j.forsciint.2016.05.018

[36] Sutton PRN (1969) Zygomatic diameter: the thickness of the tissues over the zygions. Am J Phys Anthropol 30:303–10 5772049 10.1002/ajpa.1330300215

[37] El-Mehallawi IH, Soliman EM (2001) Ultrasonic assessment of facial soft tissue thicknesses in adult Egyptians. Forensic Sci Int 117(1–2):99–107 11230951 10.1016/s0379-0738(00)00453-9

[38] Rhine JS, Campbell HR (1980) Thickness of facial tissues in American blacks. J Forensic Sci 25(4):847–858 7430993

[39] Cavanagh D, Steyn M (2011) Facial reconstruction: soft tissue thickness values for South African black females. Forensic Sci Int 206(13):215–e1 10.1016/j.forsciint.2011.01.009 21288672

[40] Phillips V, Smuts N (1996) Facial reconstruction: utilisation of computerised tomography to measure facial tissue thickness in a mixed racial population. Forensic Sci Int 83(1):51–59 8939013 10.1016/0379-0738(96)02010-5

[41] Shehata TI, Khattab N, Wahab TMA, Ekram AM, El-Bashar YH (2023) Imaging analysis of cone beam computed tomography for present Egyptians’ facial soft tissue thicknesses. J Opt 52(2):915–923

[42] De Greef S, Claes P, Mollemans W, Loubele M, Vandermeulen D, Suetens P, Willems G (2005) Semi-automated ultrasound facial soft tissue depth registration: method and validation. J Forensic Sci 50(6):JFS2004547–JFS2004547 16382819

[43] Adegbite N, Mura M, Shafiu H, Avery C, Ahmed W (2025) Forensic facial reconstruction: A computed tomography study of facial soft tissue thickness in Nigerian adult male multi-ethnic population. International Journal of Legal Medicine. 10.1007/s00414-025-03455-9 40067360 10.1007/s00414-025-03455-9 PMC12170741

[44] Atuwo AA, Bunza DB (2022) Seeing is believing: Identifying A True Hausa Man. East African Scholars Journal of Education, Humanities and Literature 5(3):87–92

[45] De Greef S, Vandermeulen D, Claes P, Suetens P, Willems G (2009) The influence of sex, age, and body mass index on facial soft tissue depths. Forensic Sci Med Pathol 5:60–65 19437147 10.1007/s12024-009-9085-9

[46] Ahmad MM, Ahmed H, Airede K (2013) Body mass index among school adolescents in Sokoto, north-western Nigeria. Sahel Med J 16(1):5–9

[47] https://www.radiantviewer.com(Accessed 14 March 2024)

[48] Lindsay KE, Rühli FJ, Deleon VB (2015) Revealing the face of ancient Egyptian: synthesis of current and traditional approaches to evidence-based facial approximation. Anat Rec 298(6):1144–1161 10.1002/ar.23146 25998648

[49] https://www.worldometers.info/demographics/life-expectancy/ (Accessed 12 September 2024)

[50] Tilotta F, Richard J, Glaunes M, Berar S, Gey S, Verdeille Y, Rozenholc JF, Gaudy (2009) Construction and analysis of a head CT-scan database for craniofacial reconstruction. Forensic Sci Int 191(1–3):112–e1 10.1016/j.forsciint.2009.06.017 19665327

[51] Toneva D, Nikolova S, Georgiev I, Harizanov S, Zlatareva D, Hadjidekov V, Lazarov N (2018) Facial soft tissue thicknesses in Bulgarian adults: relation to sex, body mass index and bilateral asymmetry. Folia Morphol 77(3):570–582 10.5603/FM.a2017.0114 29235090

[52] Hona TWPT, Stephan CN (2024) Global facial soft tissue thicknesses for craniofacial identification (2023): a review of 140 years of data since Welcker’s first study. Int J Legal Med 138(2):519–535 37804332 10.1007/s00414-023-03087-x PMC10861615

[53] Stephan CN, Preisler R, Bulut O, Bennett M (2016) Turning the tables of sex distinction in craniofacial identification: why females possess thicker facial soft tissues than males, not vice versa. Am J Phys Anthropol 161(2):283–295 27324815 10.1002/ajpa.23029

[54] Lee W-J, Wilkinson CM, Hwang H-S, Lee S-M (2015) Correlation between average tissue depth data and quantitative accuracy of forensic craniofacial reconstructions measured by geometric surface comparison method. J Forensic Sci 60(3):572–580 25739646 10.1111/1556-4029.12726

[55] Munn L, Stephan CN (2018) Changes in face topography from supine-to-upright position—and soft tissue correction values for craniofacial identification. Forensic Sci Int 289:40–50. 10.1016/j.forsciint.2018.05.016 29852367 10.1016/j.forsciint.2018.05.016

[56] Bulut O, Liu C-Y, Koca F, Wilkinson C (2017) Comparison of three-dimensional facial morphology between upright and supine positions employing three-dimensional scanner from live subjects. Leg Med 27:32–37. 10.1016/j.legalmed.2017.06.002 10.1016/j.legalmed.2017.06.002 28675828

[57] Stephan CN, Meikle B, Freudenstein N, Taylor R, Claes P (2019) Facial soft tissue thicknesses in craniofacial identification: data collection protocols and associated measurement errors. Forensic Sci Int 304:1–20. 10.1016/j.forsciint.2019.109965 10.1016/j.forsciint.2019.109965 31610333

[58] Campanelli V, Howell S, Hull M (2020) Morphological errors in 3D bone models of the distal femur and proximal tibia generated from magnetic resonance imaging and computed tomography determined using two registration methods. Comput Methods Biomech Biomed Eng Imaging Vis 8:31–39. 10.1080/21681163.2018.1559101

[59] Montagu A (1935) The location of the nasion in the living. Am J Phys Anthropol 20:81–93. 10.1002/ajpa1330200110

[60] Meikle B, Stephan CN (2020) B-mode ultrasound measurement of facial soft tissue thickness for craniofacial identification: a standardized approach. J Forensic Sci 65:939–947. 10.1111/1556-4029.14230 31671226 10.1111/1556-4029.14230

[61] Guyomarc’h P, Santos F, Dutailly B, Desbarats P, Bou C, Coqueugniot H (2012) Three-dimensional computer-assisted craniometrics: a comparison of the uncertainty in measurement induced by surface reconstruction performed by two computer programs. Forensic Sci Int 219(1–3):221–227 22297143 10.1016/j.forsciint.2012.01.008

[62] Starbuck JM, Ward RE (2007) The effect of tissue depth variation on craniofacial reconstructions. Forensic Sci Int 172(2–3):130–136 17353107 10.1016/j.forsciint.2007.01.006

